# Glucuronidation by UGT1A1 Is the Dominant Pathway of the Metabolic Disposition of Belinostat in Liver Cancer Patients

**DOI:** 10.1371/journal.pone.0054522

**Published:** 2013-01-30

**Authors:** Ling-Zhi Wang, Jacqueline Ramírez, Winnie Yeo, Mei-Yi Michelle Chan, Win-Lwin Thuya, Jie-Ying Amelia Lau, Seow-Ching Wan, Andrea Li-Ann Wong, Ying-Kiat Zee, Robert Lim, Soo-Chin Lee, Paul C. Ho, How-Sung Lee, Anthony Chan, Sherry Ansher, Mark J. Ratain, Boon-Cher Goh

**Affiliations:** 1 Cancer Science Institute of Singapore, National University of Singapore, Singapore, Singapore; 2 Department of Pharmacology, National University of Singapore, Singapore, Singapore; 3 Department of Medicine, The University of Chicago, Chicago, Illinois, United States of America; 4 Department of Clinical Oncology, Chinese University of Hong Kong, Hong Kong, China; 5 Department of Pharmacy, National University of Singapore, Singapore, Singapore; 6 Department of Hematology & Oncology, National University Health System, Singapore, Singapore; 7 Cancer Therapy Evaluation Program, Bethesda, Maryland, United States of America; 8 Committee on Clinical Pharmacology and Pharmacogenomics, The University of Chicago, Chicago, Illinois, United States of America; 9 Comprehensive Cancer Center, The University of Chicago, Chicago, Illinois, United States of America; Johnson & Johnson Medical, China

## Abstract

Belinostat is a hydroxamate class HDAC inhibitor that has demonstrated activity in peripheral T-cell lymphoma and is undergoing clinical trials for non-hematologic malignancies. We studied the pharmacokinetics of belinostat in hepatocellular carcinoma patients to determine the main pathway of metabolism of belinostat. The pharmacokinetics of belinostat in liver cancer patients were characterized by rapid plasma clearance of belinostat with extensive metabolism with more than 4-fold greater relative systemic exposure of major metabolite, belinostat glucuronide than that of belinostat. There was significant interindividual variability of belinostat glucuronidation. The major pathway of metabolism involves UGT1A1-mediated glucuronidation and a good correlation has been identified between belinostat glucuronide formation and glucuronidation of known UGT1A1 substrates. In addition, liver microsomes harboring UGT1A1*28 alleles have lower glucuronidation activity for belinostat compared to those with wildtype UGT1A1. The main metabolic pathway of belinostat is through glucuronidation mediated primarily by UGT1A1, a highly polymorphic enzyme. The clinical significance of this finding remains to be determined.

**Trial Registration:**

ClinicalTrials.gov NCT00321594

## Introduction

Histone deacetylase inhibitors (HDACis) have been shown to have anticancer activity in malignant cells through acetylation of both histone and non-histone proteins. Acetylation of histones mediates epigenetic modulation of genes that can induce apoptosis, inhibit cell growth and reduce neoangiogenesis [1]–[7]. Several HDACis have been approved for treatment of peripheral or cutaneous T-cell lymphoma [8]–[10].

Belinostat is a hydroxamic acid based pan-histone deacetylase inhibitor in clinical development as an anti-cancer therapy in a range of hematological and solid malignancies, both as single agent as well as combination therapies [11]–[18]. Currently belinostat has been granted fast track and orphan drug designation by the U.S. Food and Drug Administration for treatment of relapsed or refractory peripheral T-cell lymphoma. Although clinically the drug has been relatively well tolerated, the common adverse events included fatigue, nausea and vomiting, and lethargy, reaching grade 3 at the maximum tolerable dose of 1200 mg/m^2^ given in 30 minute (min) infusions for 5 consecutive days every 3 weeks. Belinostat is available in both oral and intravenous formulations, and as the drug is expected to be administered on a chronic basis, cumulative toxicities are possible. Therefore, it would be important to determine the metabolism of belinostat, to understand its *in vivo* disposition. Up till now, very little is known about the metabolic pathways of belinostat.

Other members of the hydroxamic acid class HDAC inhibitors like vorinostat and panobinostat undergo primary metabolism by glucuronidation [19]–[21]. On the other hand, romidepsin, a cyclic tetrapeptide class HDAC inhibitor, undergoes metabolism mainly by CYP3A4 [22]. Therefore, we hypothesized that glucuronidation would be the primary pathway for metabolism of belinostat. Furthermore, many glucuronosyl transferase isoforms are highly polymorphic and impact function, for example, homozygous deletion of UGT2B17 alleles (UGT2B17*2) results in defective metabolism of vorinostat [23]. Accordingly, it would be important to identify the main drug metabolizing isoform of belinostat, to better understand the influence of pharmacogenetics on the interindividual variability of belinostat pharmacodynamics. This also provides guidance for further drug interaction studies.

In this study, we studied the pharmacokinetics of belinostat and identified its metabolites based on the mass spectra and maximum UV absorption. We identified the dominant metabolite of belinostat as belinostat glucuronide, and further studied the glucuronidation of belinostat using a panel of UGT isoenzymes and human liver microsomes, to determine the main isoform responsible for its metabolism. Finally, potential pharmacogenetic influence on pharmacodynamics of belinostat was explored.

## Materials and Methods

The protocol for this trial is available as supporting information; see Protocol S1.

### Reagents

Belinostat (PXD101) was a gift from the National Cancer Institute (Bethesda, MD, USA). Belinostat glucuronide (belinostat-G) was chromatographically separated with HPLC–UV and isolated from human plasma using a fraction collector. Its chemical structure and purity has been verified through LC-MS/MS (API 4000 triple-quadrupole mass spectrometer; AB Sciex, Concord, Canada) and HPLC-UV analysis. Vorinostat glucuronide (vorinostat-G), the internal standard, was a gift from Merck Sharp & Dohme (I.A.) Corp. Acetonitrile (HPLC grade), methanol (HPLC grade), ethanol (analytical grade), formic acid (analytical grade), di-sodium hydrogen phosphate dihydrate (Na_2_HPO_4._2H_2_O) and orthophosphoric acid (85%) were obtained from Merck (Darmstadt, Germany). Direct-QTM water (Millipore Milford, MA, USA) was used for the mobile phase preparation. Human UGT supersomes and UGT Reaction-Solutions A and B were purchased from BD Gentest (San Jose, CA, USA). These are cDNA expressed UGTs and a panel of 12 (UGT1A1, UGT1A3, UGT1A4, UGT1A6, UGT1A7, UGT1A8, UGT1A9, UGT1A10, UGT2B4, UGT2B7, UGT2B15, UGT2B17) was used.

### Human Liver Microsome Preparation

Human livers from 37 normal Caucasian donors were processed in Dr. Mary Relling’s laboratory at St. Jude Children’s Research Hospital (Memphis, TN, USA) and were provided by the Liver Tissue Procurement and Distribution System (funded by #N01-DK-9-2310) and by the Cooperative Human Tissue Network. The 37 human liver microsomes (HLM) were isolated from different liver specimen with no correlation each other. Microsomes were prepared by differential centrifugation.

### Study Cohorts

This multicentre phase I trial of belinostat was done in Hong Kong and Singapore. All patients provided written informed consent according to Good Clinical Practice guidelines, and the protocol was approved by the institutional review boards of Prince of Wales Hospital, Hong Kong and National University Hospital, Singapore. Seventeen patients were treated with belinostat at escalating doses of 600 (n = 3), 900 (n = 3), 1,200 (n = 6), 1,400 (n = 5) mg/m^2^ daily by intravenous (i.v.) infusion over 30 min for 5 days every 21 days.

### Pharmacokinetic Assessment

Blood specimens were collected at predetermined sampling time points on day 1 at predose, 15 min, 30 min, 45 min, 1 hour (h), 1.5 h, 2 h, 3 h, 5 h and 24 h postinfusion and day 5 at predose, 30 min, 1 h, 1.5 h, 3 h, 5 h postinfusion and day 22 following the start of 30 min i.v. infusion in a phase I clinical trial of belinostat. Venous blood samples had been collected in heparinized tubes. Collected blood samples were centrifuged at 3000 g for 10–15 min and the plasma (supernatant) was separated from the cell pellet and stored in plain tubes at –80°C till analysis. The concentrations of belinostat and belinostat glucuronide were quantified by a modified high-performance liquid chromatography/mass spectrometry method [24]. Briefly, vorinostat-G was used as the internal standard for belinostat-G. The mass spectrometer was operated in positive ion mode with the optimal mass transitions of belinostat-G: *m/z* 495>93 and vorinostat-G: *m/z* 441>232, respectively. The analytical method was well validated with good linearity (coefficient of determination, r^2^≥0.999) in the range of 1–100 µM for belinostat-G.

### Identification of Belinostat Metabolites and Isolation of Belinostat-G

Metabolite screening in patient samples was processed with HPLC-UV at 268 nm, the maximum absorption wavelength of belinostat. Baseline separation of all the analytes was achieved on the Alltima C_18_ (150 mm×2.1 mm, 5 µm) column. Mobile phase solvent A was 20 mM Na_2_HPO_4_ (pH 4.2, adjusted by orthophosphoric acid), while mobile phase solvent B comprised of 70% acetonitrile and 30% methanol (v/v). A gradient mobile phase programme was used – initial percentage of solvent A was 75% (v/v) and that of solvent B was 25% (v/v); percentage of solvent B was increased to 95% (v/v) over 13 min and immediately switched back to 25% (v/v) for 7 min before injection of the subsequent sample. Total run time of each sample was 25 min. The flow rate was maintained at 0.5 mL/min.

The potential belinostat metabolites were further verified with a tandem mass spectrometer in Q1, product and precursor scans. The isolation and purification of the major metabolite of belinostat was performed on a Nova-Pak 3.9×300 mm C18 column (Waters, Ireland), using 20 mM ammonium acetate buffer (pH 5.0) and 100% methanol in an initial mobile phase composition of 60%∶40% (v/v) with a flow rate of 0.6 mL/min. Methanol was increased to 95% over 10 min and immediately switched back to 40% for 6 min before the next injection. β-Glucuronidase hydrolysis and mass spectrometry (both in positive and negative mode) to ascertain purity and identity of the major metabolite.

### Acidic Stability Test for Belinostat-G

1 mg/mL of belinostat-G and vorinostat-G was prepared in various vehicles with different pH values. These vehicles included 10 mM ammonium acetate (pH = 6.5), 10 mM ammonium formate (pH = 6.0), 0.1% acetic acid (pH = 3.2) and 0.1% formic acid (pH = 2.6). All of these test samples were incubated at 37°C for 24 h. Milli Q water (pH = 5.5) was used as control solution which was stored in −20°C. All the samples were analyzed by LC-MS/MS to obtain the values of peak area for belinostat-G and vorinostat-G. The acidic stability of both conjugated compounds was evaluated through comparison of peak area ratio of test samples versus control.

### *In Vitro* Potency Assessment on Belinostat and Belinostat-G

To determine the presence or absence of dose-dependent activity of belinostat and belinostat-G against hepatocellular cancer cells, human hepatocellular liver carcinoma (HepG2) cells were seeded in two 24-well plates at 10,000 cells per well. To one plate, 5 different concentrations of belinostat were added to obtain 2 replicates per concentration (0.1, 0.5, 1, 2, 5, 10 µM). To the other plate, equal molar concentrations of belinostat-G were added similarly. After 72 h, cells were stained with Gentian Violet paint (0.5% w/v; B.P.) and assessed for viability. Furthermore, the sensitivities of HepG2 to belinostat and belinostat-G were quantitatively measured using the MTS assay. In brief, HepG2 cells seeded in 96-well plates at 3,000 cells/well were treated with seven different doses of belinostat or belinostat-G and then assessed for growth inhibition with Promega CellTiter 96® AQ_ueous_ Non-Radioactive Cell Proliferation Assay. After incubating 24 h, 100-µL drug solution or blank medium was added into each well of 96-well plates. 72 h later, 20 µL of MTS/PES was added into each well for incubation for 3 h. OD values are measured on wavelength 490 nm. Cell growth inhibition was calculated based on the luminescent units with subtraction of background reading for only medium containing wells. The growth inhibition was defined as the percentage of the average luminescent units from drug-treated wells over that from control wells without treatment. The experiment was done in triplicates. IC_50_ values were calculated using GraphPad PRISM software (GraphPad Software, Inc., La Jolla, CA, USA).

### Western Blotting

For western blot analysis, the HepG2 cells treated with belinostat were harvested and lysed in cell lysis buffer containing 50 mM Tris HCl, pH 7.4, 150 mM NaCl, 1 mM EDTA, 1% TRITON X-100 (Sigma-Aldrich) and protease inhibitor cocktail (Roche). Protein concentrations were quantified using the Bradford assay (Invitrogen). Proteins were separated on a 12% SDS-PAGE gel and transferred to nitrocellulose membrane. Nonspecific protein binding was blocked by incubating with 5% non-fat milk (BioRad Laboratories) in 0.1% PBS-Tween (PBST) at room temperature for 1 h and incubated with specific antibodies, Histone H3 and Ac-H3 [K9/K14] (Cell Signaling), at 4°C overnight. Appropriate species-specific Horseradish peroxidises-conjugated secondary antibodies were then added and incubated at room temperature for 1 h followed by detection using an enhanced chemiluminescence kit (GE Healthcare).

### Screening of 12 Human UGT Supersomes and Kinetic Analysis

A typical incubation consisted of 50 µg of UGT, 2 mM UDPGA (UGT Reaction Solution A), 50 mM Tris-HCl, 8 mM MgCl_2_ and 0.025 mg/ml alamethicin (UGT Reaction Solution B) in a final incubation volume of 100 µL. Before the addition of substrate, the incubation mixture was prewarmed and equilibrated for 5 min at 37°C. Reactions were initiated by addition of 10 µM belinostat solution in DMSO and incubated at 37°C for 30 min; the reactions were terminated by adding 200 µL of ice-cold methanol and vortexing. The incubation mixture was vortexed and centrifuged (10,060 *g*) at 4°C for 10 min, and the supernatants were analyzed. Insect microsomes without UGT cDNA served as negative control. Kinetic parameters (*K*_m_ and *V*_max_) of belinostat glucuronidation by UGT1A1 supersomes were determined using substrate concentrations of 10 to 750 µM. Incubations were performed as described above. Three independent experiments were performed for kinetic analysis.

### Correlation Study with UGT1A1 Substrates, UGT1A1 Gene Expression and UGT1A1*28 Polymorphism

In a correlation study of glucuronidation of belinostat and UGT1A1 substrates, belinostat glucuronidation was measured as described above using 100 µM belinostat and 50 µg HLM; this concentration of belinostat was selected as it is close to the Km of UGT1A1. Glucuronidation of bilirubin, thyroxine and SN-38 in these microsomes, measurement of UGT1A1 gene expression and genotyping for UGT1A1*28 have been previously described [25]–[27].

### Data Analysis

Belinostat-G formation velocity (*v*) was calculated as Cm, 30 min/incubation time/CYP concentration, where Cm, 30 min was the metabolite concentration after 30 min incubation. Plots of substrate concentration, S (X axis) versus *v* (Y axis) were then constructed. Km and Vmax were calculated using the Michaelis-Menten equation (GraphPad software, Inc., San Diego, CA, USA).
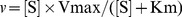
(1)


Association between belinostat glucuronide concentration and UGT1A1 substrates were performed using both Pearson’s correlation and Spearman’s correlation tests (GraphPad Prism software, La Jolla, CA). Pharmacokinetic parameters were estimated based on non-compartmental analysis model using WinNonlin software version 5.3 (Pharmsight, Sunnyvale, CA, USA). Relative exposure of belinostat-G in patient plasma was represented by the molar concentration AUC ratio of belinostat-G over belinostat. One way analysis of variance (ANOVA) was used to determine the significance among the genotypes with Tukey posthoc tests for adjustment for comparison between individual groups. A value of p<0.05 was considered to be significant.

## Results

### Pharmacokinetics and Metabolism of Belinostat in Human Plasma

**Figure 1:**
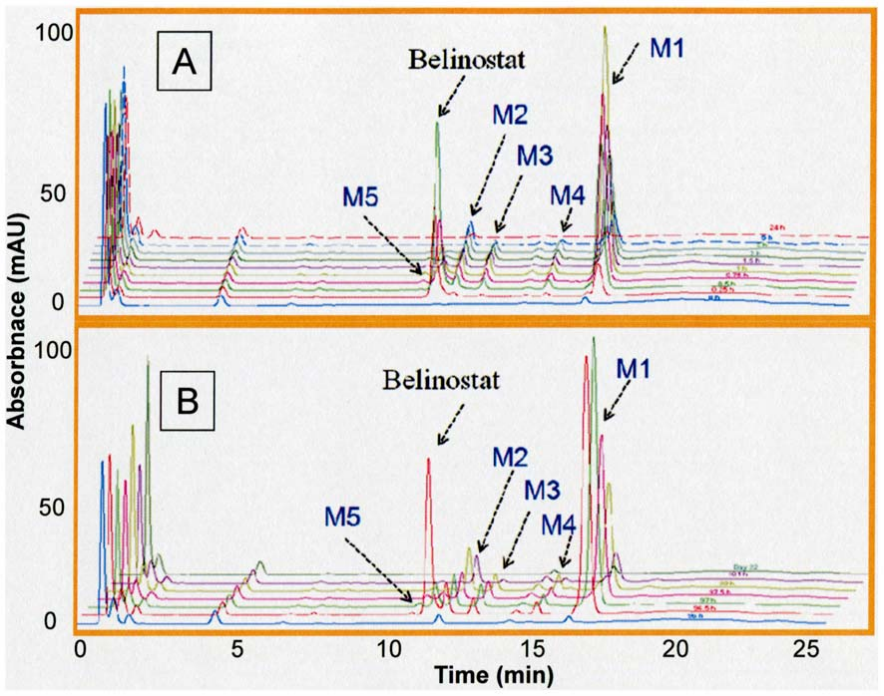
Identification of belinostat metabolites in human plasma using HPLC-UV at maximum absorption wavelength (λ = 268 nm). Chromatogram at day 5 and day 22 (A); day1(B).

The concentrations of belinostat and belinostat-G in 17 patients enrolled in a phase I clinical trial in hepatocellular carcinoma (HCC) were quantified for estimation of pharmacokinetic parameters (Table 1). Belinostat followed linear pharmacokinetics at doses ranging from 600 to 1400 mg/m^2^, with larger interindividual variability of clearance at higher doses. The coefficients of variation of Clearance (CL) were 29.0% and 30.6% at 1200 and 1400 mg/m^2^, respectively. Belinostat was eliminated rapidly after i.v. administration due to intensive phase II glucuronide conjugation, M1 (Figure 1).

**Table 1:** Pharmacokinetic parameters for belinostat after 30 min i.v. infusion in Phase I trial.

Substance	Parameter*	600 mg/m^2^	900 mg/m^2^	1200 mg/m^2^	1400 mg/m^2^
Belinostat	No. of Patients	3	3	6	5
	Cmax, µmol/L	88.2±8.5	98.5±29.7	153.5±50.4	174.4±48.3
	Tmax, h	0.33±0.14	0.42±0.14	0.64±0.10	0.40±0.14
	AUC_0–24 h_, h× µmol/L	61.3±1.2	70.0±17.3	112.6±37.1	149.0±46.2
	T_1/2_, h	3.54±0.34	4.07±0.39	4.14±0.42	3.54±0.50
	CL, L/h	52.6±3.8	70.5±17.9	54.6±15.8	53.0±16.2
	Vz, L	268.3±26.6	409.2±76.7	304.9±185.6	279.1±120.8
					
Belinostat-G	AUC_0–24 h_, h× µmol/L	286.5±36.6	302.1±54.8	514.1±41.4	575.1±154.0
Relative Exposure	Bel-G/Bel AUC ratio	4.79±0.46	4.22±0.15	4.82±1.80	4.13±0.68

Abbreviation: Cmax, maximum concentration; Tmax, time to maximum concentration; AUC_0–24 h,_ area under the curve from 0 to 24 h; T_1/2_, half-life at the elimination phase; CL, clearance; Vz, volume of distribution. *Mean ± SD; AUC ratio was calculated based on AUC_0–24 h_ of Bel-G (belinostat-G) over AUC_0–24 h_ of Bel (belinostat).

**Figure 2:**
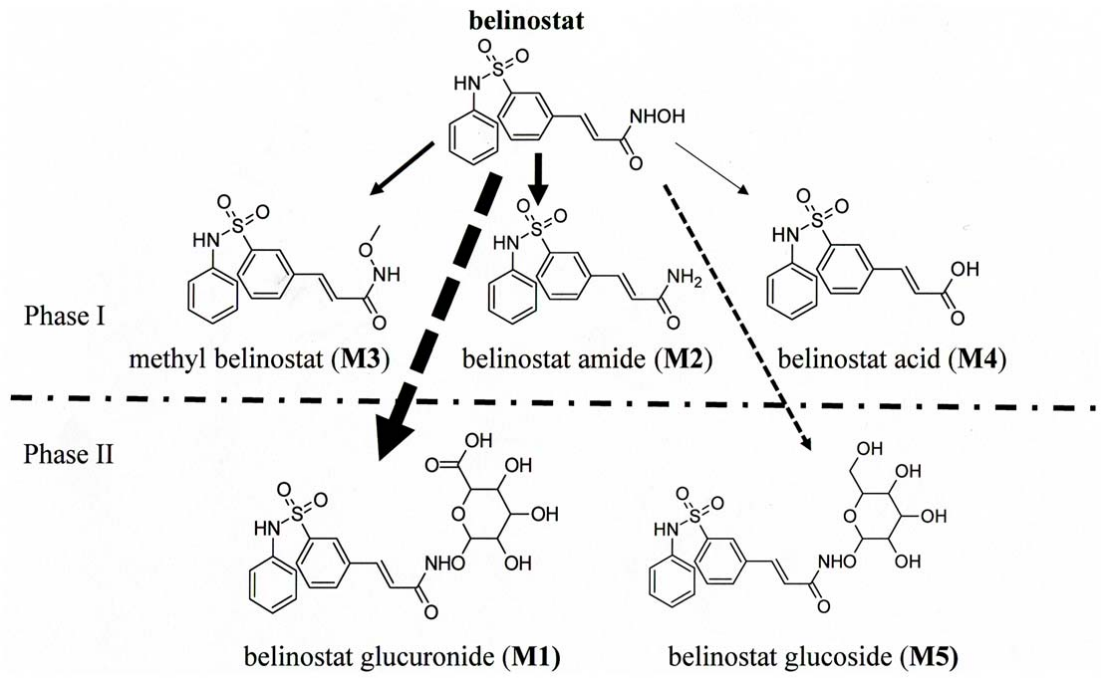
Belinostat metabolism pathway in human plasma with glucuronidation of belinostat as the dominant metabolism.

Five metabolites of belinostat were identified through comparing the ultraviolet (UV) absorption of plasma samples at 268 nm before and after dosing. Similar chromatogram profiles were detected on day 1 (Figure 1B) and day 5 (Figure 1A). The MS/MS product ions of these five metabolites monitored in positive mode supported their chemical structure identification (Table 2). Glucuronidation was the most significant pathway of belinostat metabolism; two alternate biotransformation pathways involved methylation to methyl belinostat and reduction of the hydroxamic group to its corresponding belinostat amide. Belinostat acid and belinostat glucoside were 2 other minor metabolites detected. The proposed metabolic pathway of belinostat is indicated in Figure 2. The chemical structures of these metabolites are proposed based on their protonated molecule mass peaks [M+H] **^+^**, 2-fold molecule mass peaks [2M+H] **^+^**and mass fragmentation profiling generated by product scan. These structures were further confirmed by analysis of fragmentation ions of precursor ions, which revealed a common product ion (m/z 93, phenylamino fragment) of belinostat and its five metabolites. Based on the metabolite structures, the hydroxamide moiety is the key structure for the potency of belinostat. Scanning of m/z spectra from the plasma of patients supported glucuronidation of belinostat through detection of 495, 989, 493 representing the protonated belinostat G, protonated double belinostat G, and deprotonated belinostat G, respectively. The chemical structure of belinostat-G isolated from plasma was elucidated by mass spectrometry. A series of mass spectra of belinostat-G were acquired to propose the chemical structure. For instance, m/z 495 and m/z 989 were identified as its protonated molecule mass peaks [M+H]**^+^** and 2-fold molecule mass peaks [2M+H]**^+^**, respectively. Scanning in negative mode detected its corresponding deprotonated parent ion as m/z 493[M-H]**^–^**. A similar mass fragmentation profiling in positive mode was found both for belinostat and belinostat-G. Based on the common product ion (phenylamino fragment), a precursor ion scan of m/z 93 was processed on belinostat and belinostat-G to derive their parent ions as m/z 319 [M+H]**^+^** for belinostat and m/z 495 [M+H]**^+^** for belinostat-G, respectively. Assuming this was correct, the chemical structures of its metabolites would be expected to possess similar UV spectra for these metabolites and its parent compound as well due to similarity on their basic molecular structures, and this was supported by UV spectral analysis generated by diode array detector (DAD) showing similar maximum wavelength at 268 nm for belinostat and its five metabolites.

**Table 2:** Identification of belinostat metabolites in human plasma using HPLC-DAD & LC-MS/MS analyses.

Peak ID	Retention Time (min)	λ (nm)	[M+H]^ +^	MS/MS	Proposed Structure
Belinostat	11.5	268	319	93, 204, 268, 286	Parent compound
M1	16.8	268	495	93, 204, 268, 286, 319	Belinostat glucuronide
M2	12.1	268	303	93, 204, 268, 286	Belinostat amide
M3	13.0	268	333	93, 204, 268, 286	Methyl belinostat
M4	15.1	268	304	93, 268, 286,319	Belinostat acid
M5	10.9	268	481	93, 204, 268, 286, 319	Belinostat glucoside

The major metabolite was identified as belinostat-G which was further confirmed through enzyme hydrolysis reaction. The isolated belinostat-G from patient plasma samples was subjected to *Escherichia coli-*derived β-glucuronidase (6.8 mg/mL) incubation for 5 h, and the resulting reaction mixture was analyzed through LC-MS/MS. The quantitative pharmacokinetic analysis indicated that belinostat-G exposure was 4-fold higher than that of belinostat in human plasma based on the molar concentration AUC ratio of belinostat-G/belinostat (Table 1). Hence, belinostat glucuronidation was confirmed to be the dominant metabolic pathway for belinostat.

### Stability of Belinostat-G in Acidic Environment

To further characterize the glucuronidation of belinostat, we tested the acidic stability of belinostat-G. Belinostat-G and vorinostat-G, a parallel control for belinostat-G, were demonstrated to be stable after 24 h incubation at 37°C in various acidic solutions (pH 2.6– pH 6.5). The variation in concentrations measured at the beginning of the experiment and after 24 h was very marginal (<4%). This is consistent with our proposed O-conjugated structure of belinostat-G (M1) shown in Figure 2. However, minor degradation (7%) was identified only when the pH value was decreased to 2.6. The results confirmed belinostat is conjugated to glucuronide at the O position, as O-glucuronides but not N-glucuronides of hydroxamic acids are stable in acidic environment [28], [29]. As vorinostat-G has been confirmed to be an O-conjugated glucuronide [30], it demonstrates that the isolated belinostat-G is an O-conjugated metabolite as well.

### Determination of *In Vitro* Cytotoxicity Effects of Belinostat and Belinostat-G

**Figure 3:**
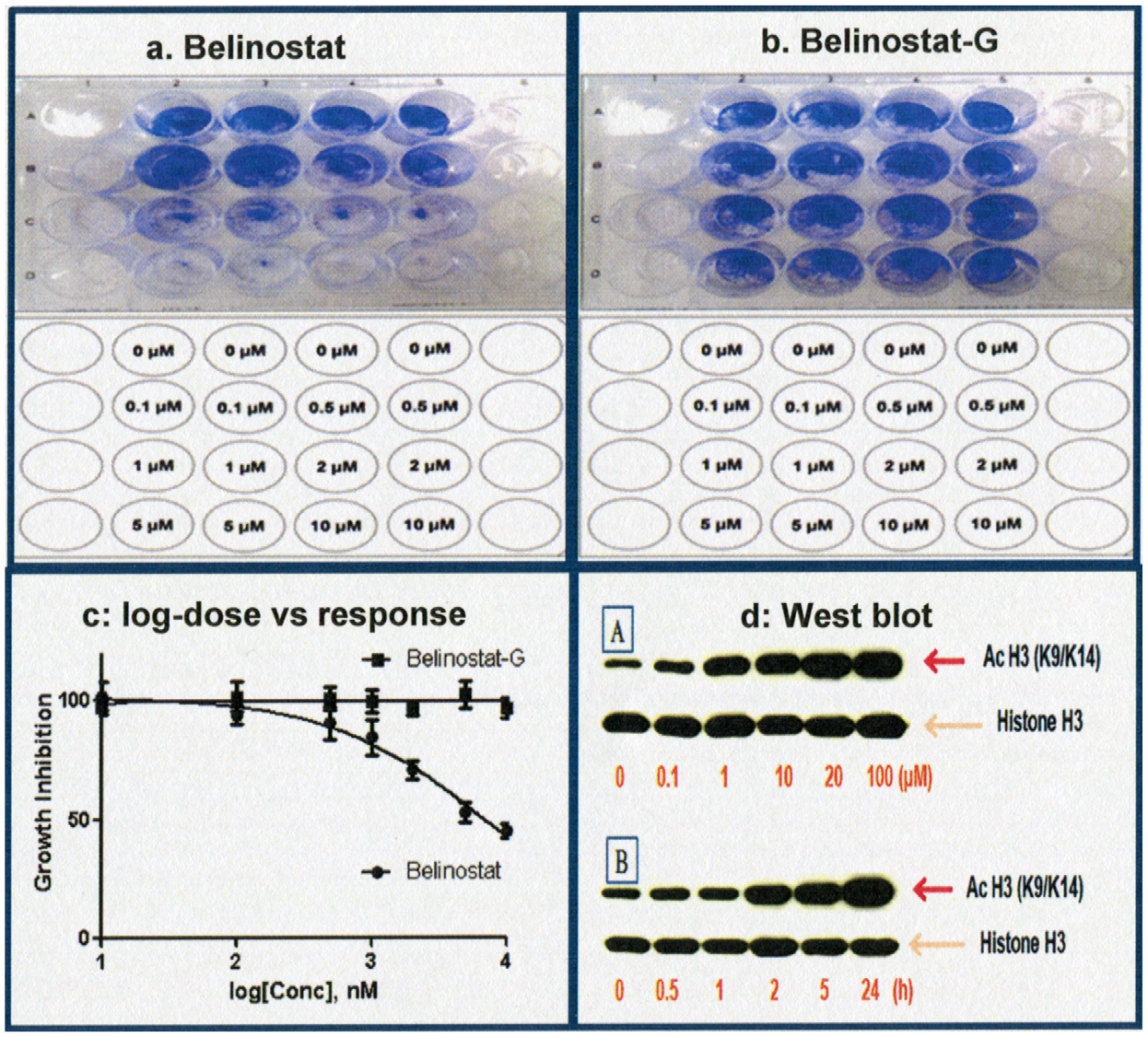
Cytotoxicity and acetylation activity on HepG2. a: belinostat incubation; b: belinostat-G incubation; (templates for concentrations added (lower) and results of 24-well dose-increasing concentrations on HepG2 Cells (upper). c: MTS results for belinostat (IC_50_ = 6.4 µM) and belinostat-G (cannot be converged). d:Belinostat acetylation activity on HepG2 cells (western blot). A: Acetyl histone 3 increased with dose increment after 5 h incubation; B: Kinetic changes of acetyl histone 3 with time increment at 10 µM.

Figure 3a and 3b show the results of dose-increasing concentrations of belinostat and belinostat-G conducted on separate 24-well plates after incubation for 72 h. As belinostat concentration increased, number of viable cells (stained violet) showed dose-dependent decrease. Conversely, increasing concentrations of belinostat-G did not seem to affect cell viability within the concentration range tested. As such, at equimolar concentrations between 0–10 µM, belinostat demonstrates significant dose-dependent cytotoxicity while belinostat-G does not. Furthermore, the results of MTS assays were also consistent with that of the staining assay (Figure 3c). Histone H3 acetylation for belinostat exposure demonstrated a time and concentration dependent increase (Figure 3d).

### *In Vitro* Metabolism Screening of UGTs for Belinostat-G Formation and Enzyme Kinetics

**Figure 4:**
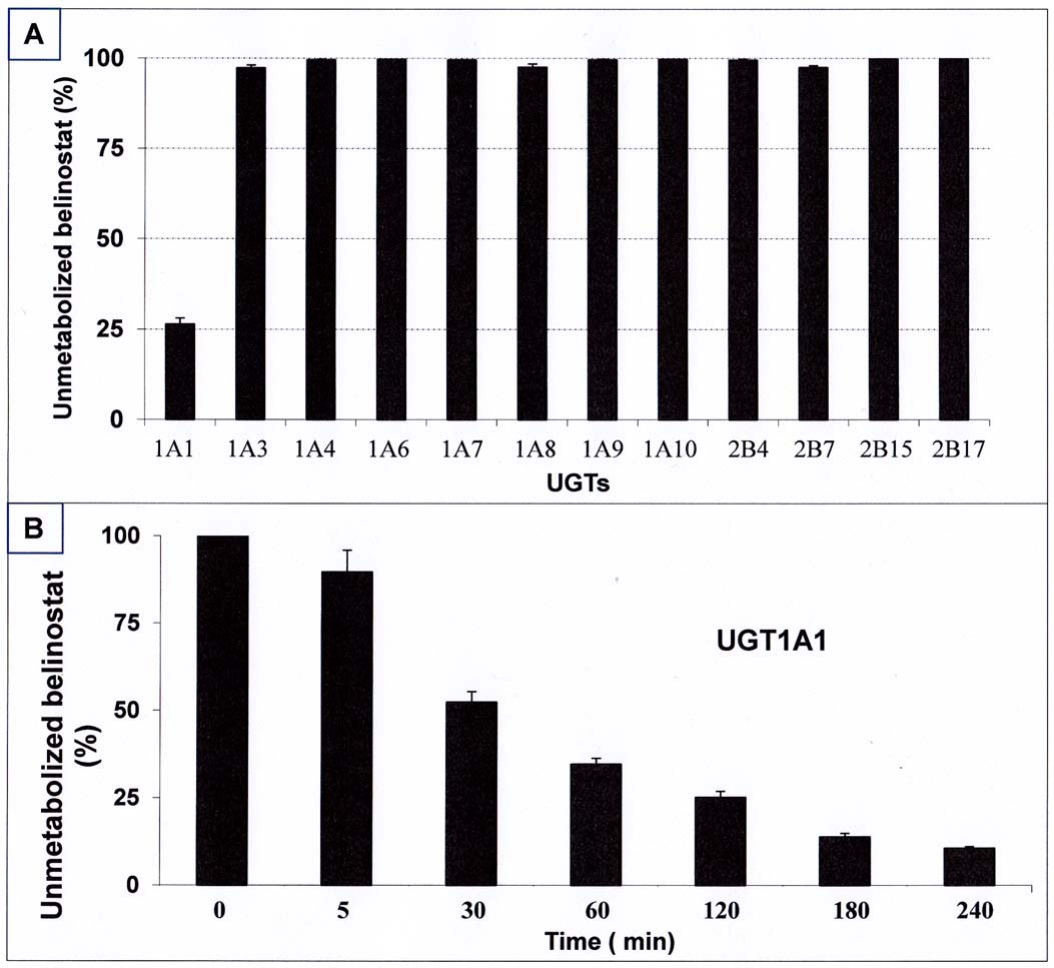
Enzyme stability test in a panel of 14 UGT isoforms after 2 h incubation at 37°C (A); Time course of glucuronidation of belinostat by UGT1A1supersomes at 37°C (B).

**Figure 5:**
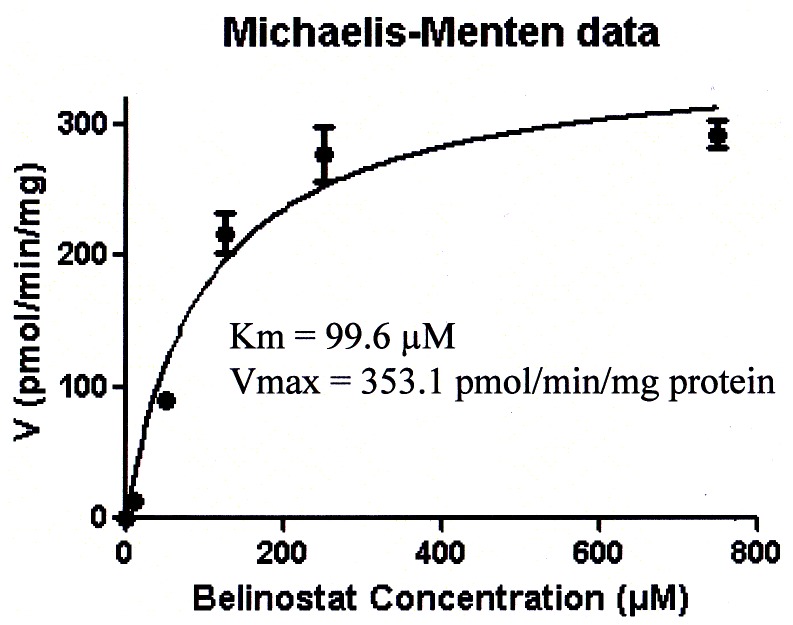
Enzyme kinetics of glucuronidation of belinostat by UGT1A1. The apparent Km and Vmax values for the glucuronide formation were 99.6 µM and 353.1 pmol/min/mg protein, respectively.

UGT1A1 glucuronidated belinostat significantly (Figure 4A), whereas no metabolism was observed after incubation with UGT1A4, UGT1A6, UGT1A7, UGT1A9, UGT1A10, UGT2B15 and UGT2B17. Minor metabolism (less than 3%) was detected with UGT1A3, UGT1A8, UGT2B4 and UGT2B7. 73.6% and 89.4% of belinostat was converted to belinostat-G after 2 h and 4 h of incubation with UGT1A1, respectively (Figure 4B). Enzyme kinetic parameters for the glucuronidation of belinostat were estimated by fitting the pooled data from the UGT1A1 incubations performed in triplicate to the Michaelis-Menten equation. The apparent Km and Vmax values for the glucuronide formation were 99.6 µM and 353.1 pmol/min/mg protein, respectively (Figure 5). The intrinsic clearance (Vmax/Km) for formation of belinostat-G was estimated to be 3.5 µL/min/mg protein. In the control insect microsomes, no belinostat-G formation was observed.

### Correlation of Glucuronidation of Belinostat with UGT Substrates

**Figure 6:**
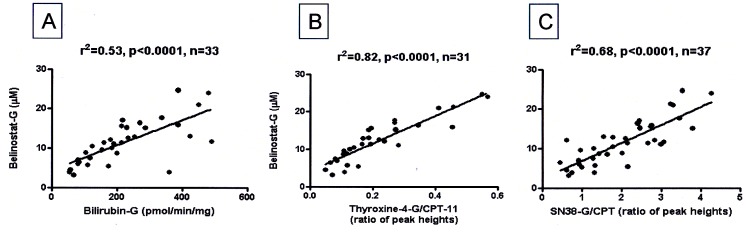
Association between glucuronidation of belinostat and UGT1A1 substrates (A, B, and C). A: Bilirubin-G; B: thyroxine-4-G/CPT-11; C: SN38-G/CPT11.

**Figure 7:**
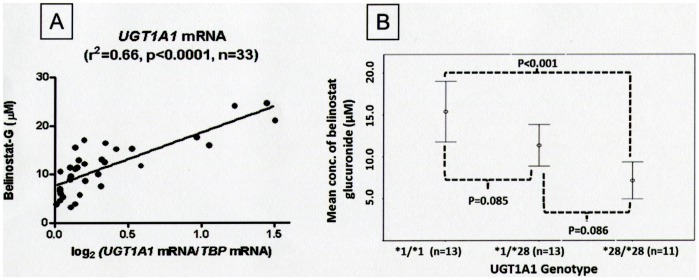
UGT1A1 expression on belinostat glucuronidation and impact of the common UGT1A1*28 promoter polymorphism. A: Correlation of belinostat glucuronide formation with UGT1A1 expression in human liver microsomes; B: Belinostat glucuronide formation by human liver microsomes according to wild-type, heterozygous and homozygous UGT1A1*28 genotypes.

In human liver microsomes, the association between glucuronidation of belinostat and UGT substrates were tested using parametric (Person’s correlation) and non-parametric (Spearman’s correlation) tests. Belinostat-G formation correlated strongly with bilirubin glucuronide formation (Figure 6A) (Pearson r^2^ = 0.53; Spearman r^2^ = 0.61 p<0.0001) and other well established substrates of UGT1A1 like thyroxine and SN38 (Pearson r^2^ = 0.68; Spearman r^2^ = 0.81 and Pearson r^2^ = 0.82; Spearman r^2^ = 0.62, respectively, all p<0.001) (Figure 6B, and 6C). Belinostat-G concentrations after incubation with the HLM correlated well with UGT1A1 mRNA expression (Pearson, r^2^ = 0.66, p<0.0001, Figure 7A). Taken together, these suggest that UGT1A1 is the predominant UGT isoform in belinostat glucuronidation.

### UGT1A1 Genotype and Glucuronidation of Belinostat in Human Liver Microsomes and Correlation with UGT1A1 Gene Expression

UGT1A1 is a polymorphic enzyme with allelic variants that influence its enzymatic activity. UGT1A1*28 is a frequent polymorphism that reduces glucuronidation activity. Therefore, to determine the effect of UGT1A1*28 on formation of belinostat-G, we measured belinostat-G concentrations after incubation of belinostat with each of 37 HLM samples previously genotyped for UGT1A1*28. The 30-min post-incubation belinostat-G concentrations (mean ± SD) were 15.39±6.00, 11.35±4.11 and 7.14±3.28 µmol for UGT1A1*1 homozygous, UGT1A1*28 heterozygous and homozygous microsomes, respectively. One way ANOVA analysis showed that significant difference between groups was detected (p = 0.01). Tukey posthoc analysis suggested that only the difference between UGT1A1*1 and UGT1A1*28 homozygous microsomes was statistically significant. As shown in Figure 7B, UGT1A1*1 homozygous microsomes had 2 fold greater mean belinostat-G concentrations compared to UGT1A1*28 homozygous microsomes after incubation (*p*<0.001).

## Discussion

To our knowledge, this is the first report describing the glucuronidation of belinostat. We have demonstrated that belinostat undergoes extensive metabolism through glucuronidation occurring at the hydroxamate moiety, leading to inactivation of its cytotoxicity. This is consistent among other hydroxamate class HDAC inhibitors as well, where glucuronidation plays a significant role in their metabolism. The metabolite profiling after incubation with a panel of supersomes overexpressing specific UGT isoforms, correlation with glucuronidation rates of known UGT1A1 substrates using human liver microsomes, and analysis of the mass spectrometry profile of belinostat in a cohort of patients with liver cancer provided evidence that belinostat glucuronidation *in vitro* and *in vivo* is mediated primarily by UGT1A1. Interestingly, the enzyme isoform that primarily metabolises another hydroxamic based HDAC inhibitor vorinostat *in vivo* is UGT2B17, whereas belinostat is metabolised by UGT1A1, which is a more abundant isoform in the human liver [31]. UGT1A1 is present in the gastrointestinal tract, which would likely reduce oral absorption of belinostat, and lead to reduced bioavailability [32]. In addition, the high efficiency of UGT1A1 for metabolising belinostat will likely reduce further its oral bioavailability through extensive first pass metabolism, posing a challenge to development of oral belinostat.

The HDAC inhibitors that are in clinical development have potential for serious side effects. Some class related effects include thrombocytopenia, prolonged QT interval on the electrocardiogram, severe fatigue and gastrointestinal side effects, and these appear to be dose dependent. Belinostat has been associated with fatigue, diarrhea and prolongation of QT interval in phase I and II trials [11], [33]. In view of its narrow therapeutic window, it is important to determine the factors such as pharmacogenetics of belinostat that account for interindividual variability of its pharmacokinetics. We previously showed that patients with deleted UGT2B17 alleles have lower vorinostat-glucuronide to vorinostat area-under-the-curve ratio and greater side effects [20]. Accordingly, it would be important to understand the extent of interindividual variability of belinostat pharmacokinetics and pharmacodynamics attributable to polymorphic expression of UGT1A1. Our study in human liver microsomes is the first step in this direction.

UGT1A1*28 is a genetic polymorphism at the promoter region where an additional TA repeat is present in the TATA box that usually has 6 TA repeats, and results in reduced expression of UGT1A1 [34]. In human liver microsomes, glucuronidation of SN-38, the active metabolite of irinotecan, is less efficient in microsomes harbouring UGT1A1*28 compared to wildtype genotype [25]. Concordantly, individuals with one or more UGT1A1*28 alleles experience higher risk of neutropenia from irinotecan treatment at doses higher than 250 mg/m^2^ [35], [36]. This polymorphism is also more common in Caucasians than Asians, with an allelic frequency of about 30% in Caucasians and 10% in Asians [37]. Our data shows that UGT1A1*28 is associated with reduced belinostat glucuronidation, which suggests that patients with this genotype may potentially have higher exposure to active belinostat resulting from impaired clearance of belinostat. Though the clinical consequences of this higher exposure with the recommended dose of belinostat are currently unknown, higher toxicities to belinostat are expected. If this is proven, UGT1A1 genotyping prior to belinostat therapy, as is currently available for irinotecan, is a way to individualise therapy.

Whether UGT1A1*28 influences the pharmacokinetics and more crucially the pharmacodynamics of belinostat in patients remains to be studied; our data supports the need for such future studies in patients. In addition, the relevance of other polymorphisms of UGT1A1 should be studied further, as there is substantial ethnogeographical variability of UGT1A1 variants. For example, UGT1A1*28 is less common in East Asians, where UGT1A1*6 (Gly71R) is more common, and associated with reduced enzymatic activity.

Given the significance of UGT1A1 in the metabolism of belinostat, it would be important to study the potential interactions of known UGT1A1 inhibitors and inducers on belinostat PK and pharmacodynamics. This is especially pertinent when belinostat is given in combination with irinotecan, a cytotoxic drug which also undergoes glucuronidation by UGT1A1 [12]. In colorectal cancer cell lines, the combination of belinostat and irinotecan was highly synergistic, and it is conceivable that such a combination is planned in patients with colorectal cancers in the near future.

## Supporting Information

Protocol S1 **Trial protocol.**
(PDF)
