# Correction: Glucuronidation by UGT1A1 Is the Dominant Pathway of the Metabolic Disposition of Belinostat in Liver Cancer Patients

**DOI:** 10.1371/annotation/df796504-a2ea-4807-a412-85985bb2550b

**Published:** 2014-01-13

**Authors:** Ling-Zhi Wang, Jacqueline Ramírez, Winnie Yeo, Mei-Yi Michelle Chan, Win-Lwin Thuya, Jie-Ying Amelia Lau, Seow-Ching Wan, Andrea Li-Ann Wong, Ying-Kiat Zee, Robert Lim, Soo-Chin Lee, Paul C. Ho, How-Sung Lee, Anthony Chan, Sherry Ansher, Mark J. Ratain, Boon-Cher Goh

There was an omission in the funding information. The correct funding information is as follows: 

"The study was sponsored by the National Research Foundation of Singapore (Experimental Therapeutics Program), the Pharmacogenomics of Anticancer Agents Research (PAAR) Group, NIH/NIGMS U01GM61393 and the National Medical Research Council of Singapore (NMRC/CSA/021/2010). The funders had no role in study design, data collection and analysis, decision to publish, or preparation of the manuscript."

