# Supplementary material for: Glucuronidation by UGT1A1 Is the Dominant Pathway of the Metabolic Disposition of Belinostat in Liver Cancer Patients
Source: PLoS One. 2013 Jan 30;8(1):e54522. doi: 10.1371/journal.pone.0054522 (PMC3559838; doi:10.1371/journal.pone.0054522)
Supplement: Protocol S1 — Trial protocol. (PDF) [file pone.0054522.s001.pdf]

Protocol version date: 16 October 2006

**NCI Protocol #:** PXD101; NSC726630 [CTEP **LOI 7237**]

**Local Protocol #:** CTRG-HC06/21/05

**TITLE:** A phase I/II study of PXD101 in patients with unresectable hepatocellular carcinoma with pharmacokinetic and pharmacodynamic evaluation

**Sponsor: The Division of Cancer Treatment and Diagnosis, National Cancer Institute, USA.**

**Coordinating Center:** Cancer Therapeutics Research Group
Comprehensive Cancer Trials Unit
Department of Clinical Oncology,
Chinese University of Hong Kong,
Prince of Wales Hospital, Shatin,
Hong Kong.
Tel: (852) 2632 2118
Fax: (852) 2648 7097

**Institutions:**
Chinese University of Hong Kong, Hong Kong.
Yonsei Cancer Center, Korea.
National University Hospital, Singapore.
Sydney Cancer Centre, Sydney, Australia.
University of Western Australia, Sir Charles Gairdner Hospital Perth, Australia.
National Cancer Centre, Singapore.
Johns Hopkins Singapore International Medial Centre, Singapore

| **Institution:** | **OHRP Assurance #:** |
|---|---|
| Chinese University of Hong Kong Prince of Wales Hospital, Shatin, Hong Kong | FWA00004034 |
| Severance Hospital, Yonsei Cancer Center, Korea | FWA00001684 |
| National University Hospital, Singapore | FWA00000205 |
| Sydney Cancer Centre, Sydney, Australia. | FWA00000341 |
| Sir Charles Gairdner Hospital, University of Western Australia, Perth, Australia. | FWA00003408 |
| National Cancer Centre, Singapore | FWA00001980 |
| Johns Hopkins Singapore International Medial Centre, Singapore | FWA00000204 |

***Principal Investigator:** Winnie **Yeo**,
Department of Clinical Oncology,

Chinese University of Hong Kong,
Prince of Wales Hospital, Shatin, Hong Kong.
Tel: (852) 2632 2118
Fax: (852) 2648 7097
E-mail: winnieyeo@cuhk.edu.hk

**Co-Investigators:**

Michael **Boyer**
Sydney Cancer Centre
Royal Prince Alfred Hospital
Missenden Road
Camperdown
Sydney, NSW 2050
Australia
Phone: +61 (2) 9515 5494
Fax: +61 (2) 9519 1546
E-mail : Michael.Boyer@cs.nsw.gov.au

Anthony TC **Chan**
Department of Clinical Oncology,
Chinese University of Hong Kong,
Prince of Wales Hospital, Shatin, Hong Kong.
Tel: (852) 2632 2118
Fax: (852) 2648 7097
E-mail: anthonytcchan@cuhk.edu.hk

Dr Alex **Chang**
Johns Hopkins Singapore International Medial Centre
11 Jalan Tan Tock Seng
Singapore 308433
Tel : +65 6880 2213
Fax: +65 6880 2220
Email address: alexchang@imc.jhmi.edu

Hyun Cheol **Chung**
Division of Haematology-Onology
Yonsei Cancer Center
Yonsei University College of Medicine
134 Shinchon-dong
Seodaemun-Ku
Seoul 120-752
Korea
Tel: +82 (2) 361 7623
Fax: +82 (2) 393 3652
E-mail: unchung8@yumc.yonsei.ac.kr

Pun **Hui**
Department of Clinical Oncology
Chinese University of Hong Kong
Prince of Wales Hospital, Shatin, Hong Kong
Tel: (852) 2632 2118
Fax: (852) 2648 7097
E-mail: huipun@hotmail.com

Lisa **Horvath**
Sydney Cancer Centre
Royal Prince Alfred Hospital
Missenden Road
Camperdown
Sydney, NSW 2050
Australia
Phone: +61 (2) 9515 5494
Fax: +61 (2) 9519 1546
E-mail: lisa.horvath@cs.nsw.gov.au

Hei Cheul **Jeung**
Yonsei metastasis research center
Yonsei Cancer Center
Yonsei University College of Medicine
134 Shinchon-dong
Seodaemun-Ku
Seoul 120-752
Korea
Tel: +82 (2) 361-7842
Fax: +82 (2) 393 3652
E-mail: jeunghc1123@yumc.yonsei.ac.kr

Robert **Lim**
National University Hospital
Dept. of Haematology-Oncology
5 Lower Kent Ridge Road
Singapore 119074
Republic of Singapore
Phone: +65 6772-4616
Fax: +65 6777-5545
E-mail: limr@nuh.com.sg

Brigette **Ma**
Department of Clinical Oncology,
Chinese University of Hong Kong,
Prince of Wales Hospital, Shatin, Hong Kong.

Tel: (852) 2632 2118
Fax: (852) 2648 7097
E-mail: brigette@clo.cuhk.edu.hk

Michael **Millward**
School of Medicine and Pharmacology
University of Western Australia
Sir Charles Gairdner Hospital
Perth
Australia
Tel: (61)-8 93463823, fax
Fax: (61)-8 93462816
E-mail: millward@cyllene.uwa.edu.au

Tony SK **Mok**
Department of Clinical Oncology,
Chinese University of Hong Kong,
Prince of Wales Hospital, Shatin, Hong Kong.
Tel: (852) 2632 2118
Fax: (852) 2648 7097
E-mail: mok206551@cuhk.edu.hk

Sun Young **Rha**
Division of Haematology-Onology
Yonsei Cancer Center
Yonsei University College of Medicine
134 Shinchon-dong
Seodaemun-Ku
Seoul 120-752
Korea
Tel: +82 (2) 361 7655
Fax: +82 (2) 393 3652
E-mail: rha7655@yumc.yonsei.ac.kr

Jae Kyung **Roh**
Division of Haematology-Onology
Yonsei Cancer Center
Yonsei University College of Medicine
134 Shinchon-dong
Seodaemun-Ku
Seoul 120-752
Korea
Tel: +82 (2) 361 7618
Fax: +82 (2) 393 3652
E-mail: jkroh@yumc.yonsei.ac.kr

Han Chong **Toh**
Department of Medical Oncology
National Cancer Centre
11 Hospital Drive
Singapore 169610
Tel +65 6436 8200
Fax +65 6227 2759
E-mail: dmothc@nccs.com.sg

Dr Wei Peng **Yong**
National University Hospital
Dept. of Haematology-Oncology
5 Lower Kent Ridge Road
Singapore 119074
Republic of Singapore
Phone: +65 6772-4670
Fax: +65 6777-5545
E-mail: wei_peng_yong@nuh.com.sg

Lam **Chan**
Department of Clinical Oncology,
Chinese University of Hong Kong,
Prince of Wales Hospital, Shatin, Hong Kong.
Tel: (852) 2632 2118
Fax: (852) 2648 7097
E-mail: l_chan@clo.cuhk.edu.hk

Han Chong **Toh**
Department of Medical Oncology
National Cancer Centre
11 Hospital Drive
Singapore 169610
Tel +65 6436 8200
Fax +65 6227 2759
E-mail: dmothc@nccs.com.sg

Dr Kian Fong **Foo**
Department of Medical Oncology
National Cancer Centre
11 Hospital Drive
Singapore 169610
Tel +65 6436 8200
Fax +65 6227 2759
E-mail: dmoteh@nccs.com.sg

**Non-Clinical/Laboratory investigators:**

Boon Cher **Goh**
National University Hospital
Dept. of Haematology-Oncology
5 Lower Kent Ridge Road
Singapore 119074
Republic of Singapore
Phone: +65 6772-4617
Fax: +65 6777-5545
E-mail: gohbc@nuh.com.sg

Qian **Tao**
Department of Clinical Oncology,
Chinese University of Hong Kong,
Prince of Wales Hospital, Shatin, Hong Kong.
Tel: (852) 2632 2118
Fax: (852) 2648 7097
E-mail: qtao@clo.cuhk.edu.hk

Simon Chung Ho **Yu**
Department of Diagnostic Radiology and Organ Imaging,
Chinese University of Hong Kong,
Prince of Wales Hospital, Shatin, Hong Kong.
Tel: (852) 2632 2285
Fax: (852) 2648 7097
E-mail: simonyu@cuhk.edu.hk

Simon SM **Ho**
Department of Diagnostic Radiology and Organ Imaging,
Chinese University of Hong Kong,
Prince of Wales Hospital, Shatin, Hong Kong.
Tel: (852) 2632 1018
Fax: (852) 2648 7097
E-mail: ssmho@cuhk.edu.hk

Ka-Fai **To**
Department of Anatomical and Cellular Pathology,
Chinese University of Hong Kong,
Prince of Wales Hospital, Shatin, Hong Kong.
Tel: (852) 2632 2361
Fax: (852) 2648 7097
E-mail: kfto@cuhk.edu.hk

**Statistician:** Benny **Zee**
Comprehensive Cancer Trials Unit
Department of Clinical Oncology,
Chinese University of Hong Kong,
Prince of Wales Hospital, Shatin, Hong Kong.
Tel: (852) 2632 1032
Fax: (852) 2632 5816
E-mail: bzee@cuhk.edu.hk

**Study Coordinator:** Jane **Koh**
Comprehensive Cancer Trials Unit
Department of Clinical Oncology,
Chinese University of Hong Kong,
Prince of Wales Hospital, Shatin, Hong Kong.
Tel: (852) 2632 1142
Fax: (852) 2947 8901
E-mail: jane@clo.cuhk.edu.hk

**Responsible Research Nurse:** Jane **Koh**
Comprehensive Cancer Trials Unit
Department of Clinical Oncology,
Chinese University of Hong Kong,
Prince of Wales Hospital, Shatin, Hong Kong.
Tel: (852) 2632 1142
Fax: (852) 2947 8901
E-mail: jane@clo.cuhk.edu.hk

**Responsible Data Manager:** Frankie KF **Mo**
Comprehensive Cancer Trials Unit
Department of Clinical Oncology,
Chinese University of Hong Kong,
Prince of Wales Hospital, Shatin, Hong Kong.
Tel: (852) 2632 1214
Fax: (852) 2632 5816
E-mail: frankiemo@cuhk.edu.hk

**NCI Supplied Agent**: PXD101 (NSC #726630)

**Protocol Version Date:** 16 October 2006

## Study Synopsis

| | |
|---|---|
| Title: | A phase I/II study of PXD101 in patients with unresectable hepatocellular carcinoma with pharmacokinetic and pharmacodynamic evaluation |
| Lead Agent supplied by NCI: | PXD101<br>NSC726630 |
| Rationale/ Hypothesis: | Hepatocellular carcinoma (HCC) is one of the commonest causes of cancer morbidity and mortality in this geographical region. It is a highly aggressive tumor with only 10% of the patients being candidates for curative surgery. For the remaining 90%, the outlook is poor with a median survival of only 8 weeks. This is mainly due to advanced disease stage at presentation, with extensive local disease and/or systemic metastasis and in most cases, consisting chronic liver diseases. Chemotherapy is the main-stay of treatment for patients with inoperable tumors. However, responses to various chemotherapeutic agents have been poor, and to date, with one of the most effective cytotoxic agent, doxorubicin, the response rate has been reported to be lower than 20%.<br>Histone acetylation has a central role in the control of gene expression, influencing transcriptional control of many genes, including tumor suppressor genes. Mechanisms that involve hypoacetylation of core nucleosome histone proteins and DNA methylation have been reported to lead to the tight coiling of chromatin, thereby silencing the expression of a variety of genes, including those implicated in the regulation of cell survival, proliferation, differentiation, and apoptosis. Inhibitors of histone deacetylase (HDAC) activity have been shown to restore expression of silenced genes by remodeling the tightly coiled chromatin, which leads to the induction of differentiation, and the subsequent apoptosis.<br>PXD101 (N-hydroxy-3-[phenylsulphamoylphenyl] acryl amide, MW = 318) is a novel, low molecular weight, HDAC inhibitor. It contains a zinc-chelating hydroxamic acid moiety that contributes to high levels of potency similar to other low molecular weight HDAC inhibitors, such as trichostatin A (TSA), oxamflatin, and suberoylanilide hydroxamic acid (SAHA). HDAC inhibitors have been also demonstrated in HCC cell lines and xenografts to induce apoptosis and tumor regression, and have anti-proliferative, anti-metastatic and anti-invasive effects.<br>An on-going phase 1 is investigating 5 daily doses of PXD101 administered as a 30-minute i.v. infusion in weeks one and four. Four patients have received doses of 150 mg/m², four patients received 300 mg/m², six patients received 600 $mg/m^2/d$ (one of the first 3 patients experienced DLT of severe fatigue), and 3 patients have received 900 $mg/m^2/d$. These doses have generally been well tolerated.<br>Data from patients who have coexisting liver impairment, as in the case of patients with HCC, in which the majority of patients have chronic liver diseases, is lacking. |

| | |
|---|---|
| | In this study, we propose to conduct a phase I study to determine dose limiting toxicity (DLT) and maximum tolerated dose (MTD) of PXD101 in patients with inoperable hepatocellular carcinoma, and to assess pharmacokinetic and pharmacodynamic profiles using serum.<br><br>Once MTD is determined, the phase II of the study will be conducted to determine efficacy of PXD101 in patients with inoperable HCC.<br><br>As an optional study, we propose to assess changes in acetylation of histone proteins, and other cellular, genetic and epigenetic induction changes in the pre- and post-treatment tumor biopsies in association with PXD101 therapy in both phases of the study. |
| Endpoints: | **Phase I portion of the study:**<br>Clinical Endpoints:<br>Primary:<br>1. To determine DLT and establish MTD of PXD101 in patients with inoperable HCC.<br>Laboratory Endpoints:<br>Primary:<br>1. To assess pharmacokinetic profiles of PXD101 in patients with HCC.<br><br>**Phase II portion of the study:**<br>Clinical Endpoints:<br>Primary:<br>1. To assess tumor response according to RECIST criteria<br><br>Exploratory Endpoints (as optional subprotocol in Phase I and II portions of the study):<br>1. To assess acetylation changes of histone proteins, induction of cell-cycle arrest or apoptosis, and activation of certain cellular genes in the pre- and post- treatment tumor biopsies in association with PXD101 therapy.<br>2. To assess epigenetic changes of peripheral blood in HCC patients during treatment with PXD101 |
| Eligibility Criteria: | • Histologically or cytologically confirmed hepatocellular carcinoma that is not amenable to curative resection<br>• ECOG performance status ≤ 2<br>• Adequate haematologic, renal and hepatic function defined as: neutrophils ≥ 1.5x $10^9$/L, platelets > 100 x $10^9$/L, serum creatinine ≤ 150 umol/l, total bilirubin ≤ 30 umol/l, albumin ≥ 28 g/l, alanine transaminases (ALT) ≤ 5.0 x ULN, alkaline phosphatase ≤ 6 x ULN, prothrombin time ≤ 4 sec of ULN, absence of clinical ascites. |

| | |
|---|---|
| Treatment plan: | In the phase 1 study, the regimen and starting dosages (level I) will be:<br>PXD101 600 mg/m2/day on day 1-5<br>Every 3 weeks<br><br>Dose limiting toxicities (DLT) is defined as any grade 4 hematological toxicity and any grade 3 or 4 nonhematological toxicity during cycle 1, excluding alopecia. Grade 3 nausea, vomiting, or diarrhea that does not respond to therapy would be considered dose-limiting. Also, delays in treatment greater than 2 weeks is also considered to be DLT.<br>The maximum tolerated dose (MTD) is defined as the dose below which $\geq$ 2 of 3 or $\geq$ 2 of 6 patients experiencing DLT. After determination of the MTD, 3 additional patients will be treated at this dose to further define toxicity.<br>At least 3 patients will be recruited to each dose level. Three additional patients (for a total of 6) will be treated at the same dose level if 1 of the first 3 exhibited DLT. Patients who experienced grade 3 nonhematological toxicity and any grade 4 hematological toxicity may continue to receive PXD101 at the next lower dose level upon resolution of all toxicities to grade 1. The drug will be discontinued for grade 4 non-hematological toxicity. For an individual patient, there could be a limit of two dose de-escalations for serious toxicity.<br>In the event that DLT occurs as defined, subsequent patients will be treated at next lower dose level.<br>Dose escalation will follow the following schema:<br>Dose levels*<br>Level -I PXD101 300 mg/m2/day on day 1-5 Every 3 weeks<br>Level I PXD101 600 mg/m2/day on day 1-5 Every 3 weeks<br>Level II PXD101 900 mg/m2/day on day 1-5 Every 3 weeks<br>Level III PXD101 1200mg/m2/day day 1-5 Every 3 weeks<br><br>Treatment will continue until disease progression or acceptable toxicities.<br>*PXD101 will be given as 30 min iv infusion through an in-line 0.22 micron low protein binding filter.<br><br>Once the MTD has been defined, a phase II study will be conducted using the MTD, with dose reduction if needed due to toxicity. |

| | |
|---|---|
| Laboratory correlates: | <u>A. To assess pharmacokinetic profiles of PXD101 in patients with HCC:</u><br>Blood samples will be collected before and following the start of infusion, with the sampling schedule being [tentative, to be refined with further info from CTEP].:<br>Day 1: 0, 15 min, 30 min, 45 min, 1 h, 1.5 h, 2 h, 3 h, 5h, 24h<br>Day 5: 0, 30 min, 1 h, 1.5 h, 3h, 5h<br>Day 22: 0 min<br><br><u>B. Optional subprotocol- To assess acetylation changes of histone proteins, induction of cell-cycle arrest or apoptosis, and activation of certain cellular genes in the pre- and post- treatment tumor biopsies in association with PXD101 therapy</u>.<br>Pre- treatment tumor biopsies and post- treatment tumor biopsies/fine needle aspiration (after 2 course of treatment at week 6 from the start of treatment) will be taken for the following laboratory studies:<br>• Hyperacetylation of histone proteins<br>• Upregulation of certain cellular genes which are not methylated in tumors: $p21^{CIP1/WAF1}$ , p27, p16 and ATM<br>• Induction of cell-cycle arrest in HCC tumor cells<br>• Changes of apoptotic index in HCC tumor cells<br><br><u>C. Optional subprotocol: Pharmacodynamic study: To assess epigenetic changes of peripheral blood in HCC patients during treatment with PXD101</u><br>Day 1: 0, 1 h, 1.5 h, 2 h, 3 h, 5h, 24h<br>Day 5: 0, 1 h, 3h, 5h<br>Day 22: 0 min |
| Statistical consideration: | Clinical Endpoints:<br><u>Phase I Portion</u><br>Objective: To determine DLT and establish MTD of PXD10. A minimum of 6 patients and a maximum of 18 patients will be required for the determination of MTD.<br><br><u>Phase II Portion</u><br>Objective: To assess if the study drug is active with respect to tumor response.<br>Statistical Considerations<br><br>The trial will be conducted in 2 stages. In the first stage, 12 patients will be accrued. If there is no response, we will stop accrual and concluded that the study treatment is uninteresting. If we have 1 or more responses, then an additional 25 patients will be accrued. |

# TABLE OF CONTENTS

Page

**SCHEMA** ................................................ i

**1. OBJECTIVES** ................................................ 1

**2. BACKGROUND** ................................................ 1
- 2.1 ***Study Disease*** ................................................ 1
- 2.2 **PXD101** ................................................ 2
- 2.3 **Rationale** ................................................ 5

**3. PATIENT SELECTION** ................................................ 6
- 3.1 **Eligibility Criteria** ................................................ 6
- 3.2 **Exclusion Criteria** ................................................ 7
- 3.3 **Inclusion of Women and Minorities** ................................................ 8

**4. REGISTRATION PROCEDURES** ................................................ 8
- 4.1 **General Guidelines** ................................................ 8
- 4.2 **Registration Process** ................................................ 8

**5. TREATMENT PLAN** ................................................ 9
- 5.1 **PXD101 Administration** ................................................ 9
- 5.2 **Supportive Care Guidelines** ................................................ 10
- 5.3 **Duration of Therapy** ................................................ 10
- 5.4 **Duration of Follow Up** ................................................ 10

**6. DOSING DELAYS/DOSE MODIFICATIONS** ................................................ 11

**7. ADVERSE EVENTS: LIST AND REPORTING REQUIREMENTS** ................................................ 13
- 7.1 **Comprehensive Adverse Events and Potential Risks List (CAEPR)** ................................................ 13
- 7.2 **Adverse Event Characteristics** ................................................ 15
- 7.3 **Expedited Adverse Event Reporting** ................................................ 15
- 7.4 **Routine Adverse Event Reporting** ................................................ 18
- 7.5 **Secondary AML/MDS** ................................................ 18

**8. PHARMACEUTICAL INFORMATION** ................................................ 18

**9. CORRELATIVE/SPECIAL STUDIES** ................................................ 20

**10. STUDY CALENDAR** ................................................ 23

**11. MEASUREMENT OF EFFECT** ................................................ 25
- 11.1 **Definitions** ................................................ 25
- 11.2 **Guidelines for Evaluation of Measurable Disease** ................................................ 26

11.3 **Response Criteria** ....................27
11.4 **Confirmatory Measurement/Duration of Response** ....................29
11.5 **Progression-Free Survival** ....................30
11.6 **Response Review** ....................30

**12. DATA REPORTING / REGULATORY CONSIDERATIONS** ....................30
12.1 **Data Reporting** ....................30
12.2 **CTEP Multicenter Guidelines** ....................31
12.3 **Cooperative Research and Development Agreement (CRADA)/ Clinical Trials Agreement (CTA)** ....................31

**13. STATISTICAL CONSIDERATIONS** ....................33

**REFERENCES** ....................35

**APPENDICES**

**APPENDIX A**
Performance Status Criteria .................... A-1

**APPENDIX B**
CTEP Multicenter Guidelines ....................B-1

**APPENDIX C**
List of medications and substances known or with the potential to interact with selected CYP450 isoenzymes ....................C-1

**APPENDIX D**
Child's-Pugh's grading for cirrhosis .................... D-1

**APPENDIX E**
Eligibility Screening Checklist ....................E-1
Patient Registration Form ....................E-2

**APPENDIX F**
Appendix F1. Sample data collection form: Pharmacokinetics....................F-1
Appendix F2. Sample data collection form: Tumor tissue assessment ....................F-2
Appendix F3. Sample data collection form: Pharmacodynamics....................F-3

**APPENDIX G**
Central Review of ECG tracings that are associated with QTc Prolongation .................... G-1

# 1. OBJECTIVES

1.1. **Phase I portion of the study:**

Clinical Endpoints:

Primary:

1. To determine DLT and establish MTD of PXD101 in patients with inoperable HCC.

Laboratory Endpoints:

Primary:

1. To assess pharmacokinetic profiles of PXD101 in patients with HCC.

1.2 **Phase II portion of the study:**

Clinical Endpoints:

Primary:

1. To assess tumor response according to RECIST criteria

1.3 **Exploratory Endpoints (as optional subprotocol in Phase I and II portions of the study):**

Laboratory Endpoints:

Secondary:

1. To assess acetylation changes of histone proteins, induction of cell-cycle arrest or apoptosis, and activation of certain cellular genes in the pre- and post- treatment tumor biopsies in association with PXD101 therapy.
2. To assess epigenetic changes of peripheral blood in HCC patients during treatment with PXD101of Pharmacodynamic study

# 2. BACKGROUND

## 2.1 *Study Disease*

Hepatocellular carcinoma (HCC) is one of the commonest causes of cancer morbidity and mortality in Asia (Parker, 1984). It is a highly aggressive tumor with only 10% of the patients being candidates for curative surgery (Okuda, 1980; Lai, 1995). For the remaining 90%, the outlook is poor with a median survival of only 8 weeks (Pawarode, 1998; Calvet 1990). This is mainly due to advanced disease stage at presentation, with extensive local disease and/or systemic metastasis and in most cases, consisting chronic liver diseases. Chemotherapy is the main-stay of treatment for patients with inoperable tumors. However, responses to various chemotherapeutic agents have been poor, and to date, with one of the most effective cytotoxic agent, doxorubicin, the response rate has been reported to be lower than 20% (Johnson, 2002; Leung, 2001).

## 2.2 PXD101

PXD101 (N-hydroxy-3-[phenylsulphamoylphenyl] acryl amide, MW = 318) is a novel, achiral, low molecular weight inhibitor of histone deacetylase (HDAC) activity (Finn *et al.*, 2002; Plumb *et al.*, 2003). The structural design of PXD101 was based on natural products that were known inhibitors of HDAC (Kalvinsh *et al.*, 2002). It contains a zinc-chelating hydroxamic acid moiety that contributes to high levels of potency similar to other low molecular weight HDAC inhibitors, such as trichostatin A (TSA), oxamflatin, and suberoylanilide hydroxamic acid (SAHA) (Marks *et al.*, 2000). Molecular modeling studies predict that PXD101 binds within the active sites of HDAC class I and II enzymes, in a similar manner to that described for TSA and SAHA (Finnin *et al.*, 1999; Investigator's Brochure, 2004).

**Mechanism of Action**

HDACs exert their action during post-translational acetylation of core nucleosomal histones, which affects chromatin structure and thus regulates gene expression. Acetylation and deacetylation of histones is controlled by the activity of histone acetyltransferases (HATs) and HDACs. Acetylation of the e-amino groups of lysine residues in the N-terminal tails of histones is associated with transcriptional activation, while deacetylation is associated with condensation of chromatin and transcriptional repression. HDAC inhibitors, such as the natural product TSA and SAHA, induce the expression of genes associated with cell cycle arrest and tumor suppression (Yoshida *et al.*, 1990; Richon *et al.*, 1998; Kelly *et al.*, 2001). Phenotypic changes induced by HDAC inhibitors include G1 and G2/M cell cycle arrest and apoptosis in tumor cells. There are also data to suggest that HDAC inhibitors possess antiangiogenic properties. HDAC inhibitors deplete vascular endothelial cell growth factor (VEGF) concentrations and inhibit the proliferation of endothelial cells (Johnstone, 2002).

DNA methylation and hypoacetylation of core nucleosome histone proteins lead to the tight coiling of chromatin, thus silencing the expression of a variety of genes (Jones and Baylin, 2002). HDAC inhibitors alone or in combination with DNA hypomethylating agents, such as 5-azacytidine or decitabine, restore expression of silenced genes leading to cell differentiation and subsequent cell cycle arrest or apoptosis. Reports also suggest that HDAC inhibitors repress some genes, *e.g.,* the gene for thymidylate synthetase, which may explain the reports of synergistic interaction between 5-FU and these inhibitors (Di Gennaro *et al.*, 2003; Glaser *et al.*, 2003). Gene expression analyses with human HL-60 promyelocytic leukemia cells and HCT116 colon cancer cells, after exposure to PXD101 (1 μM), TSA (1 μM), or FK228 (10 nM), indicate that the three HDAC inhibitors possess similar gene expression profiles in both cell lines (Investigator communication) and the data suggest that PXD101 may have similar gene induction and repression effects as other HDAC inhibitors. HDAC inhibition also depleted the levels of several oncoproteins that are normally stabilized by binding to the 90 kD heat shock protein (Hsp90) in cancer cells, *e.g.,* mutant p53 and Raf-1 proteins (Yu *et al.*, 2002). Concurrent administration of marginally toxic concentrations of 17-AAG, an Hsp90 inhibitor, with sublethal concentrations of HDAC inhibitors resulted in synergistic induction of apoptosis (Rahmani *et al.*, 2003).

## Nonclinical activity

PXD101 induced a concentration-dependent increase in acetylation of histones H3 and H4 in various tumor cell lines (ovarian, colon, lung, breast, prostate, and melanoma), with $IC_{50}$ values varying from about 10 to 100 nM (Plumb *et al.*, 2003). In these same tumor cell lines, PXD101 induced apoptosis or was cytotoxic with $IC_{50}$ values varying from 0.2 to 3.4 μM. Other human tumor cell lines reported to be sensitive to PXD101 are T-cell leukemia (Jurkat); small cell lung cancer (SCLC) (H69, GLC2, and GLC4); and multiple myeloma (JNN-2, LP-1, RPMI-8226, and U-266) (Investigator's Brochure, 2004).

The antitumor activity of PXD101 was demonstrated in several *in vivo* xenograft models, including human A2780 ovarian cancer cells, cisplatin-resistant A2780/cp70, and HCT116 colon cancer cells; and murine P388 leukemia cells (Plumb *et al.*, 2003; Investigator's Brochure, 2004). Tumor-bearing mice were treated intraperitoneally (IP) with PXD101 once daily for 7 days. Delay of tumor growth was observed at a dose of 10 mg/kg/day in A2780 and A2780/cp70 xenografts. Growth delay was dose-dependent up to a PXD101 dose of 40 mg/kg/day. Growth inhibition was also observed in xenografts of HCT116 cells at 40 mg/kg/day. PXD101 treatments did not affect body weight of the mice, nor were there apparent signs of toxicity. Hyperacetylated histone H4 was detected in peripheral blood mononuclear cells (PBMC) at 1 and 2 hours after a single IP injection of 40 mg/kg PXD101 (Plumb *et al.*, 2003), and acetylation returned to baseline levels by 3 hours. Histone hyperacetylation was dose-dependent, with marked acetylation in both PBMCs and tumor tissue apparent at doses of PXD101 ≥ 10 mg/kg.

The efficacy of various schedules of PXD101 was evaluated in the murine P388 IP tumor model (Investigator's Brochure, 2004). A 5-day-schedule 40 mg/kg/day × 5 days (from day 3) proved superior to the same total dose (200 mg/kg) administered in a bolus on day 3 ($P<0.0001$). Fractionation of the daily dose (either 20, 40, or 80 mg/kg for 5 days) into two doses per day instead of one daily dose did not improve survival. Mice given a cumulative dose of 200 mg/kg over a 10-day period (day 3 to day 12) fractionated as 20 mg/kg/day for 10 days exhibited better survival than mice receiving 40 mg/kg every two days (day 3, 5, 7, 9, 11) ($P=0.009$). Treatment every second day three times was compared with the same cumulative dose of 270 mg/kg PXD101 administered every day in a 5-day period (day 3 to 7) and no significant differences were found in survival rates ($p=0.49$). In general, daily doses appeared to be superior to bolus injections; fractionation of the daily dose into two did not result in any advantage; and experiments comparing alternating day to daily treatments favored the daily treatment, but not substantially.

## Nonclinical Pharmacology and Toxicology

Due to the low intrinsic aqueous solubility of PXD101, the early *in vivo* studies were primarily carried out using a co-solvent formulation of ethanol and polyethylene glycol

fractions (PEG) in Tris buffer (known as the co-solvent-Tris formulation) (Investigator's Brochure, 2004). However, this preparation proved unsuitable due to injection site reactions and a new formulation was developed which utilized the solubility enhancing effects of arginine and led to the identification of the PXD101 50 mg/ml injection formulation. The *in vitro* studies were carried out using PXD101 in a final concentration of <1% DMSO, while the *in vivo* studies used the new injection formulation, after demonstration of bioequivalence.

The cardiovascular effects of PXD101 were examined in dogs. The high dose (35 mg/kg) of PXD101 elicited a small transient increase in heart rate (Investigator's Brochure, 2004). Anticipated changes in the electrocardiogram waveform - such as decrease in the RR, PR and QT intervals - were also observed. However, these changes were not substantially different from the control group. Increases in the rate of respiration following administration of the intermediate (15 mg/kg) and the high (35 mg/kg) doses of PXD101 were not significantly different from those observed with the control group. The hemolytic potential and plasma compatibility of PXD101 (in the arginine formulation) in human blood were examined *ex vivo*. No hemolysis was detected.

Pharmacokinetic (PK) studies were undertaken in mice, rats and dogs (Investigator's Brochure, 2004). The majority of the metabolite profiling studies, coupled with preliminary investigation of excretion routes, were undertaken in rats and dogs. Plasma concentrations of PXD101 in mice, determined after a single IP injection of 20 mg/kg, reached a mean concentration of 3.3 ± 0.7 μM after 0.5 hour, and decreased to 0.042 ± 0.002 μM by 2 hours. In general, the $C_{max}$ was achieved at the first time point after the end of infusion and increased with dose. It then declined very rapidly with a $t_{½}$ of 0.5 to 1 hour. With daily administration, $t_{½}$ did not vary from day 1 to day 5. With few exceptions, the PK parameters did not vary from day 1 to day 5 (or 7) of a treatment cycle and did not vary from cycle 1 to cycle 2 where two cycles were administered over 4 weeks. There was no clear indication of drug accumulation following 5 or 7 days of treatment. Acetylation of histones H3 and H4 was also determined in canine PBMCs taken at various times after a 45 minute intravenous (IV) infusion of 50 mg/kg PXD101 (Plumb *et al.*, 2003). At the end of the infusion, the plasma concentration of PXD101 was between 20 and 30 μM, and the plasma $t_{½}$ was estimated as approximately 40 minutes.

In an exploratory *in vivo* study, metabolic profiling was undertaken in pooled samples from mouse, rat and dog plasma following administration of PXD101. The study suggested that rapid and extensive metabolism took place, producing a variety of metabolites that differed in rodents and dogs. Since there was no consistency of either dose or sampling time, no further conclusions could be drawn. The overall metabolic profile observed in the two species appeared to be similar. The principal degradation product and a secondary metabolite were tested in the HDAC biochemical and cell proliferation assays and found to be inactive. PXD101 did not inhibit the five major cytochrome P450 enzymes. Elimination of PXD101 and its metabolites occurred by both

urinary and fecal routes in both rats and dogs. Concentrations of parent drug cleared by either route were low when calculated as a percentage of the original dose, implying that the bulk of parent drug is cleared following primary and secondary metabolic conversion.

Toxicology studies were performed with both formulations of PXD101. Overall, in rats, the maximum tolerated dose (MTD) was considered to be between 100 and 200 mg/kg/day. Due to local irritation at injection sites, it was not possible to assign a no-observable-effect-level (NOEL) in rats. Systemic effects, however, even at the 100 mg/kg/day level, were mild with both formulations. Signs of local irritation at the injection sites with both formulations were observed in dogs as well, although a NOEL of 10 mg/kg/day was assigned in one study. Systemic and local irritation adverse events (AEs), such as lymphopenia, lymphoid atrophy, and injection site reactions, were observed at a dose of 50 mg/kg/day but these were considered mild and fully reversible for the arginine formulation.

**Phase 1 Clinical Experience**

A phase 1 study of PXD101 is currently underway, treating patients with five daily doses of PXD101 administered as a 30-minute IV infusion at weeks one and four, with doses ranging from 150 – 1200 mg/m$^2$. These doses have generally been well tolerated, with grade 1/2 fatigue, nausea, and vomiting occurring in most patients. Other frequent, mild AEs include diarrhea, constipation, and headache. This study has so far demonstrated PK parameters in humans comparable to those found in dogs. The $t_{1/2}$ and $C_{max}$ values at the different dose levels are as follows: at 150 mg/m$^2$, 46.6 ± 8.7 min and 6565 ± 2158 ng/mL, respectively; at 300 mg/m$^2$, 44.6 ± 8.0 min and 15505 ± 6245 ng/mL, respectively; at 600 mg/m$^2$, 43.4 ± 6.7 min and 31177 ± 8968 ng/mL, respectively; at 900 mg/m$^2$, 54.2 ± 8.7 min and 53779 ± 6381 ng/mL, respectively; at 1200 mg/m$^2$, 85.5 ± 19.6 min and 52362 ± 12724 ng/mL, respectively. One instance of tumor lysis syndrome was observed in a patient with multiple myeloma, who had been administered PXD101 1000 mg/m$^2$/day for 5 days. The symptoms were accompanied by decreasing renal function, increase in serum carbamide, creatinine, urate, potassium, phosphate and decrease in platelet counts. However, there was tumor response evidenced by a drop in M-component from 55 g/L to 40 g/L. The patient also experienced increasing confusion, with EEG demonstrating epileptic status. Tumor lysis syndrome with accompanying decrease in renal function was thought to be related to PXD101. The epileptic episode was considered unrelated. Stable disease has been observed in four patients with a duration of up to 3 months in two patients.

### 2.3 Rationale

Histone acetylation has a central role in the control of gene expression, influencing transcriptional control of many genes, including tumor suppressor genes. Mechanisms that involve hypoacetylation of core nucleosome histone proteins and DNA methylation have been reported to lead to the tight coiling of chromatin, thereby silencing the expression of a variety of genes, including those implicated in the regulation of cell

survival, proliferation, differentiation, and apoptosis. Inhibitors of histone deacetylase (HDAC) activity have been shown to restore expression of silenced genes by remodeling the tightly coiled chromatin, which leads to the induction of differentiation, and the subsequent apoptosis.

## 3. PATIENT SELECTION

### 3.1 Eligibility Criteria

3.1.1 Patients must have histologically or cytologically confirmed hepatocellular carcinoma that are not amenable to curative resection.

3.1.2 Patients must have measurable disease, defined as at least one lesion that can be accurately measured in at least one dimension (longest diameter to be recorded) as ≥20 mm with conventional techniques or as ≥10 mm with spiral CT /MRI scan. See section 11.2 for the evaluation of measurable disease.

3.1.3 Age ≥18 years. Because no dosing or adverse event data are currently available on the use of PXD101 in patients <18 years of age, children are excluded from this study but will be eligible for future pediatric single-agent trials, if applicable.

3.1.4 Life expectancy of greater than 12 weeks.

3.1.5 ECOG performance status ≤2 (Karnofsky ≥60%; see Appendix A).

3.1.6 Patients must have normal organ and marrow function as defined below:

| | | |
|---|---|---|
| X | leukocytes | ≥3,000/mcL |
| X | absolute neutrophil count | ≥1,500/mcL |
| X | platelets | ≥100,000/mcL |
| X | total bilirubin | ≤ 30 umol/l |
| X | albumin | ≥ 28 g/l |
| X | ALT(SGPT) | ≤ 5.0 X institutional upper limit of normal |
| X | alkaline phosphatase | ≤ 6 x ULN |
| X | prothrombin time | ≤ 4 sec above ULN |
| X | creatinine | ≤ 150 umol/l |
| X | absence of clinical ascites or encephalopathy | |

3.1.7 In vitro study of PXD101 has so far not shown PXD inhibiting the five major cytochrome P450 enzymes. Eligibility of patients receiving any medications or substances which may potentially affect the activity or pharmacokinetics of PXD101 will be determined following review of their case by the Principal Investigator. (A list of medications and substances known or with the potential to interact with selected CYP450 isoenzymes is provided in Appendix C)

3.1.8 The effects of PXD101 on the developing human fetus at the recommended therapeutic dose are unknown. For this reason and because HDAC inhibitors are known to be teratogenic, women of child-bearing potential and men must agree to use adequate contraception (hormonal or barrier method of birth control; abstinence) prior to study entry and for the duration of study participation. Should a woman become pregnant or suspect she is pregnant while participating in this study, she should inform her treating physician immediately.

3.1.9 Ability to understand and the willingness to sign a written informed consent document.

## 3.2 Exclusion Criteria

3.2.1 Child's-Pugh's grading for cirrhosis C (Appendix D)

3.2.2 Patients who have had chemotherapy or radiotherapy within 4 weeks (6 weeks for nitrosoureas or mitomycin C) prior to entering the study or those who have not recovered from adverse events due to agents administered more than 4 weeks earlier.

3.2.3 Patients may not be receiving any other investigational agents.

3.2.4 Patients with known brain metastases should be excluded from this clinical trial because of their poor prognosis and because they often develop progressive neurologic dysfunction that would confound the evaluation of neurologic and other adverse events.

3.2.5 History of allergic reactions attributed to compounds of similar chemical or biologic composition to PXD101

3.2.6 Patients should not have taken valproic acid, another histone deacytelase inhibitor, for at least 2 weeks prior to enrollment.

3.2.7 A marked baseline prolongation of QT/QTc interval, e.g., repeated

demonstration of a QTc interval >500 msec; Long QT Syndrome; the required use of concomitant medication on PXD101 infusion days that may cause Torsade de Pointes. (See Table below for list).

*Table: Drugs Associated with Prolongation of QT/QTc Interval*

From: Roden DM. Drug-induced prolongation of the QT interval. N Engl J Med 2004;350(10):1013-22

| Table 1. Drugs That May Cause Torsade de Pointes.* |
|---|
| **Drugs commonly involved** |
| Disopyramide |
| Dofetilide |
| Ibutilide |
| Procainamide |
| Quinidine |
| Sotalol |
| Bepridil |
| **Other drugs†** |
| Amiodarone |
| Arsenic trioxide |
| Cisapride |
| Calcium-channel blockers: lidoflazine (not marketed in the United States) |
| Antiinfective agents: clarithromycin, erythromycin, halofantrine, pentamidine, sparfloxacin |
| Antiemetic agents: domperidone, droperidol |
| Antipsychotic agents: chlorpromazine, haloperidol, mesoridazine, thioridazine, pimozide |
| Methadone |

* Further information on the strength of the evidence linking various drugs to torsade de pointes may be found at http://www.torsades.org.

† The level of risk associated with these drugs depends on the dose and the population being treated; in general, the risk is probably less than 1 percent.

3.2.8 Significant cardiovascular disease including unstable angina pectoris, uncontrolled hypertension, congestive heart failure related to primary cardiac disease, a condition requiring anti-arrhythmic therapy, ischemic or severe valvular heart disease, or a myocardial infarction within 6 months prior to the trial entry

3.2.9 Uncontrolled intercurrent such as, but not limited to, ongoing or active infection, or psychiatric illness/social situations that would limit compliance with study requirements.

3.2.10 Pregnant women are excluded from this study because PXD101 is an HDAC inhibitor with the potential for teratogenic or abortifacient effects. Because there is an unknown but potential risk for adverse events in nursing infants

secondary to treatment of the mother with PXD101, breastfeeding should be discontinued if the mother is treated with PXD101.

HIV-positive patients on combination antiretroviral therapy are ineligible because of the potential for pharmacokinetic interactions with PXD101. In addition, these patients are at increased risk of lethal infections when treated with marrow-suppressive therapy. Appropriate studies will be undertaken in patients receiving combination antiretroviral therapy when indicated.

### 3.3 Inclusion of Women and Minorities

Both men and women and members of all races and ethnic groups are eligible for this trial.

## 4. REGISTRATION PROCEDURES

### 4.1 General Guidelines

Eligible patients will be entered on study centrally at the Comprehensive Cancer Trials Unit, Department of Clinical Oncology, Chinese University of Hong Kong, Prince of Wales Hospital, Shatin, Hong Kong, by the Study Coordinator. All sites should call the Study Coordinator, Tel: (852) 2632 1142 Fax: (852) 2947 8901, to verify agent availability. The required forms (Eligibility Screening Checklist, Patient Registration Form) can be found in Appendix (Appendix E).

Following registration, patients should begin protocol treatment within 72 hours. Issues that would cause treatment delays should be discussed with the Principal Investigator. If a patient does not receive protocol therapy following registration, the patient's registration on the study may be canceled. The Study Coordinator should be notified of cancellations as soon as possible.

Except in very unusual circumstances, each participating institution will order DCTD-supplied agents directly from CTEP. Agents may be ordered by a participating site only after the initial IRB approval for the site has been forwarded by the Coordinating Center to the CTEP PIO (PIO@ctep.nci.nih.gov) except for Group studies.

### 4.2 Registration Process

To register a patient, the following documents should be completed by the research nurse or data manager and faxed (852) 2947 8901 or e-mailed jane@clo.cuhk.edu.hk to the Study Coordinator:

- Copy of required laboratory tests including histology report
- Signed patient consent form
- Eligibility Screening Checklist, Patient Registration Form.

The research nurse or data manager at the participating site will then call (852) 2632 1142 or e-mail jane@clo.cuhk.edu.hk the Study Coordinator to verify eligibility. To complete the registration process, the Coordinator will

- assign a patient study number
- register the patient on the study
- fax or e-mail the patient study number and dose to the participating site
- contact [preferably by email] the research nurse or data manager at the participating site and confirm registration.

## 5. TREATMENT PLAN

### 5.1 PXD101 Administration

Treatment can be administered an either as inpatient/outpatient basis. Reported adverse events and potential risks are described in Section 7. Appropriate dose modifications for PXD101 are described in Section 6. No investigational or commercial agents or therapies other than those described below may be administered with the intent to treat the patient's malignancy.
PXD101, will be added to 250 mL of 5% dextrose in water or 0.9% sodium chloride and administered via a venous catheter for 30 minutes through an in-line 0.22 micron low protein binding filter. on days 1 to 5 every 3 weeks.
Please refer to Section 8 for details on any special precautions or warnings relevant for investigational study agent administration (e.g., incompatibility of the agent with commonly used intravenous solutions, necessity of administering agent with food, premedications, etc.).

### 5.2 General Concomitant Medication and Supportive Care Guidelines

Because there is a potential for interaction of PXD101 with other concomitantly administered drugs through the cytochrome P450 system, the case report form must capture the concurrent use of all other drugs, over-the-counter medications, or alternative therapies. The Principal Investigator should be alerted if the patient is taking any agent known to affect or with the potential to affect selected P450 isoenzymes.

Treat diarrhea promptly with appropriate supportive care, including loperamide. Instruct patients to begin taking loperamide at the first signs of: 1) poorly formed or loose stool, 2) occurrence of more bowel movements than usual in one day, or 3) unusually high volume of stool. Loperamide should be taken in the following manner: 4 mg following the first episode and then 2 mg following each new episode until recovery of diarrhea (no more than 16 mg daily). Loperamide should not be taken prophylactically. Advise patients to drink plenty of clear fluids to help prevent dehydration caused by diarrhea. Avoid loperamide if there is the presence of blood

or mucus in the stool or if diarrhea is accompanied by fever.

For patients known to have chronic hepatitis B virus infection (hepatitis B surface antigen –HbsAg positive), lamivudine will be administered within 1 week prior to study treatment.

## 5.3 Duration of Therapy

In the absence of treatment delays due to adverse event(s), treatment may continue for until one of the following criteria applies:

- X Disease progression,
- X Intercurrent illness that prevents further administration of treatment,
- X Unacceptable adverse event(s),
- X Patient decides to withdraw from the study, or
- X General or specific changes in the patient's condition render the patient unacceptable for further treatment in the judgment of the investigator.

## 5.4 Duration of Follow Up

Patients will be followed for 8 weeks after 'off study' or until death, whichever occurs first [this is defined as 'end of study']. Patients who are 'off study' for unacceptable adverse events will be followed until resolution or stabilization of the adverse event. Patients who are alive after the defined 'end of study' period should be continued to follow up, either through clinic visit or via phone contact, every 2 months until death.

# 6. DOSING DELAYS/DOSE MODIFICATIONS

In the phase 1 study, the regimen and starting dosages (level I) will be:
PXD101 600 mg/m2/day on day 1-5
PXD101, will be added to 250 mL of 5% dextrose in water or 0.9% sodium chloride and administered via a venous catheter (peripheral or central) for 30 minutes.
Every 3 weeks

**Dose escalation schema for patients accrued**
Dose limiting toxicities (DLT) is defined as any grade 4 hematological toxicity and any grade 3 or 4 nonhematological toxicity during cycle 1, excluding alopecia.

Specifically, grade 3 nausea, vomiting, or diarrhea that does not respond to therapy are considered dose-limiting. Also, delays in treatment greater than 2 weeks is also dose-limiting.

At least 3 patients will be recruited to each dose level. Three additional patients (for a total of 6) will be treated at the same dose level if 1 of the first 3 exhibited DLT.
In view of the fact that HCC patients often have associated chronic liver disease, in the event a patient experiences a DLT which is considered unrelated to study drug, as agreed upon by the main investigator of the site and the principal investigator of the study, then the protocol will still enroll an additional 3 patients for a total of 6 patients in the dosing cohort. However, once the 6 patients are completed within the dosing cohort, those DLTs which are determined to be unrelated to the study drug will not be counted with those being used to define that dose that elicits a Dose Limiting Toxicity.
The maximum tolerated dose (MTD) is defined as the dose below which ≥ 2 of 3 or ≥ 2 of 6 patients experiencing DLT. After determination of the MTD, 3 additional patients will be entered at the MTD level if only 3 patients were treated previously at this dose to further define toxicity.
Patients who experienced grade 3 nonhematological toxicity and any grade 4 hematological toxicity may continue to receive PXD101 at the next lower dose level upon resolution of all toxicities to grade 1. The drug will be discontinued for grade 4 non-hematological toxicity.

Dose escalation will follow the following schema:
Dose levels*
Level -I PXD101 300 mg/m2/day on day 1-5 Every 3 weeks
Level I PXD101 600 mg/m2/day on day 1-5 Every 3 weeks
Level II PXD101 900 mg/m2/day on day 1-5 Every 3 weeks
Level III PXD101 1200mg/m2/day day 1-5 Every 3 weeks

Once the MTD has been defined with 6 patients having 0 or 1 DLTs related to the study drug, additional patients will be added up to reach the targeted number of patients for stage 1 of the phase 2 study [i.e. 12] [Please refer to section 13 for statistical design of the study]. For patients treated with MTD, dose reduction can be conducted if needed due to toxicity.

**Dose modification for subsequent courses for individual patient**

Subsequent courses of treatment will be modified according to the following rules:
1. Patients may not be retreated unless the ANC is ≥1.5 x $10^9$/L and the platelet count is ≥100 x $10^9$/L.
2. For a non-hematologic toxicity during the previous cycle, treatment may resume if the toxicity has subsided to baseline (except for alopecia) or ≤ grade 1, whichever is less restrictive.
3. Adjust the dose level based on the highest grade of toxicity observed during the previous cycle:
Grade 0-2—same PXD101 dose level

Grade 3-4—decrease PXD101 dose by one dose level
(i.e., decrease dose by 300 mg/m$^2$/day)

Treatment may be delayed for up to 2 weeks beyond planned resumption of the next cycle. If a patient does not fulfill retreatment criteria by that time, they will be removed from the trial.

If dose reduction is necessary, there will be no escalation of the PXD101 dose following recovery from toxicities. There could be a limit of two dose de-escalations for patients who experience serious toxicity.

**Dose modification for QTc prolongation**

**(a) Dose modification for QTc prolongation during a treatment cycle**

In the event of a QTc prolongation interval >500 ms on a local automated post-treatment ECG assessment during a treatment cycle, PXD101 treatment should be temporarily withheld until the QTc interval returns below 500 ms. PXD101 treatment may then be resumed at a 25% reduced dose for the remainder of the cycle. All available ECG tracings should be retained for subsequent review by a central laboratory (to be arranged by CuraGen as described in Appendix G) for a blinded digital manual analysis.

**(b) Dose modification for QTc prolongation for subsequent courses**

In cases where the QTc prolongation is confirmed by central laboratory read, the patient should receive treatment at a 25% reduced dose for all remaining cycles. If central laboratory analysis indicates that there was no evidence of QTc prolongation, treatment may be resumed at the original dose pre dose reduction.

In the even of a subsequent prolongation of QTc interval >500 ms following two dose reductions, further treatment with PXD101 should be permanently discontinued. This guidance to investigators is summarized in the table below.

| **Management of treatment-emergent grade 3 and 4 prolonged QTc interval** | |
|---|---|
| Adverse event | Action |
| Grade 3 QTc prolongation (QTc > 500ms) | Interrupt until resolved to grade 2 or less, then resume with a 25% dose reduction. Submit all available ECGs to central laboratory. If the event is confirmed by central read: maintain reduced dose. If the event is refuted by central read: resume treatment at prior dose |
| Grade 4 QTc prolongation (QTc >500 ms with life-threatening signs or symptoms (arrhythmia, CHF, hypotension, shock, syncope) or Torsades de pointes) | Discontinue treatment permanently |

| | |
|---|---|
| Recurrence of grade 3 QTc prolongation after treatment at two dose reductions | Discontinue treatment permanently |

All patients on and off therapy who develop signs and symptoms suggestive of asymptomatic QTc prolongation should be thoroughly evaluated and closely monitored and supported as clinically dictated.

## 7. ADVERSE EVENTS: LIST AND REPORTING REQUIREMENTS

Adverse event monitoring and reporting is a routine part of every clinical trial. The following list of adverse events (Section 7.1) and the characteristics of an observed adverse event (Section 7.2) will determine whether the event requires **expedited** (via AdEERS; Section 7.3) or **routine** (via CDUS; Section 12.1.1) reporting.

### 7.1 Comprehensive Adverse Events and Potential Risks List (CAEPR)

The Comprehensive Adverse Event and Potential Risks list **(**CAEPR) was developed to provide a single, complete list of reported and/or potential adverse events associated with an agent using a uniform presentation of adverse events by body system. In addition to the comprehensive list, the subset of those events that are "expected" [i.e., the Agent Specific Adverse Event List (ASAEL)] is presented in a separate column and identified with ***bold*** and ***italicized*** text. This subset is used to guide expedited reporting requirements.

**Version 2.0, June 16, 2005[1]**

| Adverse Events with Possible Relationship to PXD101 (CTCAE v3.0 Term) [n=39 patients] | | | "Agent Specific Adverse Event List" (ASAEL) |
|---|---|---|---|
| **Likely (>20%)** | **Less Likely (≤20%)** | **Rare but Serious (<3%)** | |
| CARDIAC ARRHYTHMIA | | | |
| | Atrial fibrillation | | |
| | Palpitations | | |
| | Supraventricular tachycardia | | |
| CONSTITUTIONAL SYMPTOMS | | | |
| Fatigue (asthenia, lethargy, malaise) | | | |
| | Fever (in the absence of neutropenia, where neutropenia is defined as ANC <1.0 x 10e9/L) | | |
| GASTROINTESTINAL | | | |
| | Anorexia | | |
| | Constipation | | |

| | | | |
|---|---|---|---|
| | Diarrhea | | |
| Nausea | | | |
| Vomiting | | | |
| PAIN | | | |
| | Pain - head/headache | | |
| PULMONARY/UPPER RESPIRATORY | | | |
| | Dyspnea (shortness of breath) | | |
| VASCULAR | | | |
| | Phlebitis (including superficial thrombosis) | | |

[1] This table will be updated as the toxicity profile of the agent is revised. Updates will be distributed to all Principal Investigators at the time of revision. The most current version can be obtained by contacting ADEERSMD@tech-res.com. Your name, the name of the investigator, the protocol, and the agent should be included in the e-mail.

**Also reported on PXD101 trials but with the relationship to PXD101 still undetermined:**

**Cardiac Arrhythmia** – mild QTc increases (there is a potential of asymptomatic QTc prolongation)
**Constitutional Symptoms** – rigors/chills; sweating
**Dermatology/Skin** – flushing; injection site reaction
**Gastrointestinal** – taste alteration
**Infection** – infection
**Pain –** chest pain; joint pain
**Pulmonary/Upper Respiratory** – cough
**Syndromes** – tumor lysis syndrome
**Vascular** – thrombosis

**Animal Data: The following toxicities have been observed in animal studies with PXD101:** decreased blood parameters (leukocytes, lymphocytes, hemoglobin); increased blood parameters (platelets, reticulocytes); decreased weight gain; lip licking; salivation; hemorrhagic urine; injection site hemorrhage/edema; atrophy of lymphoid organs/decreased lymphoid cellularity; unsteadiness on feet; ataxia; head shaking; prostration; subdued mood/hyperactivity; tremor; uncoordinated movements; partially closed eyelids; rapid breathing

**Note**: PXD101 in combination with other agents could cause an exacerbation of any adverse event currently known to be caused by the other agent, or the combination may result in events never previously associated with either agent.

## 7.2 Adverse Event Characteristics

- **CTCAE term (adverse event description) and grade:** The descriptions and grading scales found in the revised NCI Common Terminology Criteria for Adverse Events (CTCAE) version 3.0 will be utilized for adverse event reporting. All appropriate treatment areas should have access to a copy of the CTCAE

version 3.0. A copy of the CTCAE version 3.0 can be downloaded from the CTEP web site (http://ctep.cancer.gov/reporting/ctc.html).

- **"Expectedness"**: Adverse events can be "Expected" (see in Sect. 7.1 above) or unexpected. ***Bold and italicized*** terms in Sect. 7.1 identify expected events. See Section 7.3.4 for guidelines for reporting both types of events.

- **Attribution** of the adverse event:
  - Definite – The adverse event *is clearly related* to the study treatment.
  - Probable – The adverse event *is likely related* to the study treatment.
  - Possible – The adverse event *may be related* to the study treatment.
  - Unlikely – The adverse event *is doubtfully related* to the study treatment.
  - Unrelated – The adverse event *is clearly NOT related* to the study treatment.

## 7.3 Expedited Adverse Event Reporting

7.3.1 Expedited adverse event reporting for this study is via AdEERS (Adverse Event Expedited Reporting System), accessed via the secure CTEP web site https://webapps.ctep.nci.nih.gov/openapps/plsql/gadeers_main$.startup). The reporting procedures to be followed are presented in the "NCI Guidelines: Expedited Adverse Event Reporting Requirements for NCI Investigational Agents" which can be downloaded from the CTEP web site (http://ctep.cancer.gov/reporting/adeers.html).

7.3.2 Adverse events that require notification to IDB within 24 hours should be made via the AdEERS web site: https://webapps.ctep.nci.nih.gov/openapps/plsql/gadeers_main$.startup.

7.3.3 All adverse events reported via AdEERS **must** be copied to the Study Coordinator jane@clo.cuhk.edu.hk using the copy feature of AdEERS. The Study Coordinator will submit adverse event reports to the Principal Investigator for timely review.

7.3.4 Expedited Reporting Guidelines –

7.3.4.1 Phase 1 Trials Utilizing an Agent under a CTEP IND: AdEERS Reporting Requirements for Adverse Events That Occur Within 30 Days[1] of the Last Dose of the Investigational Agent

| | **Grade 1** | **Grade 2** | **Grade 2** | **Grade 3** | | **Grade 3** | | **Grades 4 & 5[2]** |
|---|---|---|---|---|---|---|---|---|
| | **Unexpected and Expected** | **Unexpected** | **Expected** | **Unexpected** | | **Expected** | | **Unexpected and Expected** |
| | | | | **with Hospitalization** | **without Hospitalization** | **with Hospitalization** | **without Hospitalization** | |
| **Unrelated Unlikely** | Not Required | Not Required | Not Required | 10 Calendar Days | Not Required | 10 Calendar Days | Not Required | 24-Hour; 5 Calendar Days |

| | | | | | | | | |
|---|---|---|---|---|---|---|---|---|
| **Possible Probable Definite** | Not Required | 10 Calendar Days | Not Required | 24-Hour; 5 Calendar Days | 24-Hour; 5 Calendar Days | 10 Calendar Days | Not Required | 24-Hour; 5 Calendar Days |

[1] **Adverse events with attribution of possible, probable, or definite that occur <u>greater</u> than 30 days after the last dose of treatment with an agent under a CTEP IND require reporting as follows:**
AdEERS 24-hour notification followed by complete report within 5 calendar days for:

- Grade 3 unexpected events with hospitalization or prolongation of hospitalization
- Grade 4 unexpected events
- Grade 5 expected events and unexpected events

[2] Although an AdEERS 24-hour notification is not required for death clearly related to progressive disease, a full report is required as outlined in the table.

December 15, 2004

**Note: All deaths on study require both routine and expedited reporting regardless of causality. Attribution to treatment or other cause must be provided.**

- Expedited AE reporting timelines defined:
  - "24 hours; 5 calendar days" – The investigator must initially report the AE via AdEERS within <u>24 hours</u> of learning of the event followed by a complete AdEERS report within <u>5 calendar days</u> of the initial 24-hour report.
  - "10 calendar days" - A complete AdEERS report on the AE must be submitted within <u>10 calendar days</u> of the investigator learning of the event.

- Any medical event equivalent to CTCAE grade 3, 4, or 5 that precipitates hospitalization (or prolongation of existing hospitalization) must be reported regardless of attribution and designation as expected or unexpected with the exception of any events identified as protocol-specific expedited adverse event reporting exclusions.

- Any event that results in persistent or significant disabilities/incapacities, congenital anomalies, or birth defects must be reported via AdEERS if the event occurs following treatment with an agent under a CTEP IND.

- Use the NCI protocol number and the protocol-specific patient ID provided during trial registration on all reports.

7.3.4.2 Phase 2 and 3 Trials Utilizing an Agent under a CTEP IND: AdEERS Reporting Requirements for Adverse Events That Occur Within 30 Days[1] of the Last Dose of the Investigational Agent

| | **Grade 1** | **Grade 2** | **Grade 2** | **Grade 3** | | **Grade 3** | | **Grades 4 & 5[2]** | **Grades 4 & 5[2]** |
|---|---|---|---|---|---|---|---|---|---|
| | **Unexpected and Expected** | **Unex-pected** | **Expected** | **Unexpected** | | **Expected** | | **Unex-pected** | **Expected** |
| | | | | **with Hospitali-zation** | **without Hospitali-zation** | **with Hospitali-zation** | **without Hospitali-zation** | | |

| | | | | | | | | | |
|---|---|---|---|---|---|---|---|---|---|
| **Unrelated Unlikely** | Not Required | Not Required | Not Required | 10 Calendar Days | Not Required | 10 Calendar Days | Not Required | 10 Calendar Days | 10 Calendar Days |
| **Possible Probable Definite** | Not Required | 10 Calendar Days | Not Required | 10 Calendar Days | 10 Calendar Days | 10 Calendar Days | Not Required | 24-Hour; 5 Calendar Days | 10 Calendar Days |

[1] **Adverse events with attribution of possible, probable, or definite that occur greater than 30 days after the last dose of treatment with an agent under a CTEP IND require reporting as follows:**

AdEERS 24-hour notification followed by complete report within 5 calendar days for:

- Grade 4 and Grade 5 unexpected events

AdEERS 10 calendar day report:

- Grade 3 unexpected events with hospitalization or prolongation of hospitalization
- Grade 5 expected events

[2] Although an AdEERS 24-hour notification is not required for death clearly related to progressive disease, a full report is required as outlined in the table.

December 15, 2004

**Note: All deaths on study must be reported using expedited reporting regardless of causality. Attribution to treatment or other cause should be provided.**

- Expedited AE reporting timelines defined:
  - "24 hours; 5 calendar days" – The investigator must initially report the AE via AdEERS within 24 hours of learning of the event followed by a complete AdEERS report within 5 calendar days of the initial 24-hour report.
  - "10 calendar days" - A complete AdEERS report on the AE must be submitted within 10 calendar days of the investigator learning of the event.

- Any medical event equivalent to CTCAE grade 3, 4, or 5 that precipitates hospitalization (or prolongation of existing hospitalization) must be reported regardless of designation as expected or unexpected and attribution with the exception of events identified as protocol-specific expedited adverse event reporting exclusions.

- Any event that results in persistent or significant disabilities/incapacities, congenital anomalies, or birth defects must be reported via AdEERS if the event occurs following treatment with an agent under a CTEP IND.

- Use the NCI protocol number and the protocol-specific patient ID provided during trial registration on all reports.

## 7.4 Routine Adverse Event Reporting

Those adverse events that do not require expedited reporting **must** be reported in routine (CDUS; see Section 12.1) study data submissions. **Adverse events reported through AdEERS must also be reported in routine study data submissions.**

### 7.5 Secondary AML/MDS

Investigators are required to report cases of secondary AML/MDS occurring on or following treatment on NCI-sponsored chemotherapy protocols using the NCI/CTEP Secondary AML/MDS Report Form. *This form can be downloaded from the CTEP web site (http://ctep.cancer.gov/reporting/index.html).* Second malignancies and non-AML/MDS secondary malignancies (*e.g.*, endometrial cancer in a breast cancer patient receiving tamoxifen) should NOT be reported via AdEERS but should be submitted as part of the study results via routine CDUS reporting.

## 8. PHARMACEUTICAL INFORMATION

**PXD101 (NSC #726630)**

**Chemical Name:** N-hydroxy-3-(phenylsulphamoylphenyl) acrylamide

**Classification:** Antineoplastic

**CAS Registry Number:** 414864-00-9

**Molecular Formula:** $C_{15}H_{14}N_{2}0_{4}S$ **M.W.:** 318.349

**Approximate Solubility:** Water 0.14 mg/mL; ethanol >200 mg/mL; polyethylene glycol 400 ~ 1.5 mg/mL; 1,2-propanediol ~ 0.2 mg/mL
There is a possibility of elevated subvisual particulate levels in PXD101 IV solutions at an 8.1 mg/mL concentration. Post filtration studies with an in-line 0.22 micron low protein binding filter demonstrated an insignificant effect on total drug delivered.

**Description:** Histone deacetylase (HDAC) inhibitor

**Mode of Action:** HDACs are a family of enzymes that regulate chromatin remodeling and gene transcription via the dynamic process of acetylation and deacetylation of core histones.

PXD101 is a novel and potent HDAC inhibitor of the hydroxymate class. It alters acetylation levels of histone and non-histone proteins, thus influencing chromatin accessibility and ultimately gene transcription. Additionally, recent data reveals that HDAC inhibitors reduce vascular endothelial growth factor (VEGF) production and directly inhibit the endothelial cells proliferation.

**Drug Interactions:** PXD101 is weak to moderate inhibitor of CYP2C8 and CYP2C9 and a moderate inducer of CYP1A2. Additionally, CYP3A4 contributes to PXD101 metabolism.

**How Supplied:** PXD101 is supplied by CuraGen Corporation and distributed by the CTEP, DCTD, NCI. PXD101 Injection is supplied in 10 mL vials containing 50 mg/mL of a clear yellow, sterile solution for IV administration. Each 10 mL vial contains 500 mg of PXD101, 1000 mg of L-arginine and sterile water. Vials contain approximately 0.5 mL overfill. L-arginine used in the PXD101 formulation is of non-animal origin. The product is supplied in single use Type I glass vials with ETFE-coated chlorobutyl rubber stoppers and "flip-off" aluminum crimp seals.

**Preparation:** Withdraw the calculated dose from the PXD101 vial and add to 250 mL of 5% dextrose in water or 0.9% sodium chloride.

**Storage:** Store intact vials of PXD101 in the refrigerator, 2 to 8 ºC (36 to 46 ºF). Intact vials are unstable at room temperature with a degradation amounting to about 5% at 20 weeks. Although PXD-101 displays no obvious light instability, leave vials in the secondary packaging until preparation of the dilution.

**Stability:** Shelf life stability studies of intact vials of PXD101 are on-going.

Once further diluted in 250 mL of 5% dextrose in water or 0.9% sodium chloride, PXD101 may be stored at ambient room temperature (15 - 25°C) for not more than 12 hours prior to infusion.

**Route of Administration:** Intravenous

**Method of Administration:** Infuse PXD101 intravenously over 30 minutes through an in-line 0.22 micron low protein binding filter.

**Adverse Events and Potential Risks**

A list of the adverse events and potential risks associated with PXD101 can be found in Section 7.1.

**Availability**

PXD101 is an investigational agent supplied to investigators by the Division of Cancer Treatment and Diagnosis (DCTD), NCI.

PXD101 is provided to the NCI under a Clinical Trials Agreement (CTA) between CuraGen Corporation and the DCTD, NCI (see Section 12.3).

**Agent Ordering**

NCI-supplied agents may be requested by the Principal Investigator (or their authorized designees) at each participating institution. Pharmaceutical Management Branch (PMB)

policy requires that the agent be shipped directly to the institution where the patient is to be treated. PMB does not permit the transfer of investigational agents between institutions (unless prior approval from PMB is obtained). Completed Clinical Drug Requests (NIH-986) should be submitted to the PMB by fax (301) 480-4612 or mailed to the Pharmaceutical Management Branch, CTEP, DCTD, NCI, 9000 Rockville Pike, EPN Rm. 7149, Bethesda, MD 20892.

**Agent Accountability**

The Investigator, or a responsible party designated by the Investigator, must maintain a careful record of the inventory and disposition of all agents received from DCTD using the NCI Drug Accountability Record Form. *See the CTEP web site for Policy and Guidelines for Accountability and Storage of Investigational Drugs (http://ctep.cancer.gov/requisition/storage.html).*

## 9. CORRELATIVE/SPECIAL STUDIES (HC06-1)

Histone deacetylases (HDAC) are enzymes involved in the regulation of chromatin structure changes and epigenetic regulation of gene expression. There is also a link between the epigenetic silencing of tumor suppressor genes (TSGs) (e.g. such as promoter CpG island hypermethylation) and histone deacetylation. Both play important roles in the onset and progression of cancer.

HDAC inhibitors have been shown to reactivate expression of certain cellular genes and inhibit cancer growth in pre-clinical models. PXD101 is a zinc-chelating hydroxamic acid inhibitor of class I and II HDAC, which has potent anti-tumor activity in nanomolar concentration in a variety of epithelial cancer cell lines.

Hepatocellular carcinoma (HCC) is the most frequently occurring liver carcinoma world-wide. In Asia, over 80% of HCC is etiologically associated with hepatitis B virus (HBV)-related cirrhosis. Prognosis of unresectable or metastatic HCC is poor and effective therapy is urgently needed. Epigenetic mechanisms such as promoter CpG island methylation that result in upregulation of mitogenic pathways, are thought to contribute to HCC carcinogenesis. Studies have shown that hypermethylation of tumor suppressor genes or genes that regulate key cellular processes such as apoptosis, cell cycle and DNA-damage repair are common in HCC. Furthermore, HDAC inhibitors such as trichostatin A and butyrates have been shown to induce growth inhibition, cell cycle arrest and apoptosis in HCC cell lines. The effect of PXD101 in HCC in vivo is unknown in humans.

**Objectives of the correlative studies:**

**Primary:**

1. To assess pharmacokinetic profiles of PXD101 in patients with HCC.

**Secondary:**

1. To assess acetylation changes of histone proteins, induction of cell-cycle arrest or apoptosis, and activation of certain cellular genes in the pre- and post- treatment HCC tumor

biopsies in association with PXD101.

**Study A. To assess pharmacokinetic profiles of PXD101 in patients with HCC**

Venous blood samples (5ml) were taken at various times for measurement of PXD101 from the contralateral arm (as listed below). Samples for pharmacokinetic study will be transferred to the National University hospital, Singapore.

Blood samples will be collected before and following the start of infusion of cycle 1 of treatment, with the sampling schedule being:

**Day 1: 0, 15 min, 30 min, 45 min, 1 h, 1.5 h, 2 h, 3 h, 5h, 24h**

**Day 5: 0, 30 min, 1 h, 1.5 h, 3h, 5h**

**Day 22: 0 min**

Please refer to Appendix F1, for sample collection form.

Venous blood samples should be collected in heparinized bottle. Collected samples will be centrifuged at 3000g for 10-15 minutes and the plasma separated from the cell pellet and stored in plain tubes at –80 C till analysis. For centres that require supply of bottles, please contact investigator in Charge: Dr Boon-Cher Goh.

Shipping instructions of samples:

- A fax of the inventory sheet should be sent to the attention of Mr Wang Lingzhi, Department of Pharmacology, National University of Singapore, the day before shipment of samples. The fax number is 65 67730579
- Samples and inventory sheet must be shipped by overnight delivery in a styrofoam container and packaged in dry ice to ensure that they remain frozen. Shipment must be scheduled for weekdays only, and weekends will be avoided.

**SEND SAMPLES TO:** Attn: A/Prof Lee How-Sung, PhD
HPLC Laboratory Level 4
Department of Pharmacology
MD2 National University of Singapore
Singapore 119074

**Methods- Estimation of Plasma Drug Concentrations**

An assay method for the analysis of plasma PXD101 drug levels will be performed based on high performance liquid chromatographic separation coupled with tandem LC-MS-MS detection. In summary, samples will be mixed with water:methanol:formic acid (90:10:1) and an internal standard (oxamflatin) and applied to a Waters Oasis MAX 30-mg cartridge using solid phase extraction (Gilson 215). Elution was performed with methanol:water:formic acid (90:10:2), and the extract dried and reconstituted in 200 µl of water:acetonitile:acetic acid (65:35:1) for injection. Samples will be analyzed on an Applied Biosystems API2000 triple quadrupole system through a C18(2) 50 x 3-mm column using a mobile phase consisting of water:acetonitile:acetic acid (65:35:1) with a the flow rate was 0.5 ml/min. Peak area resolution will be utilized to derive calibration curves for each run, incorporating quality control samples. Pharmacokinetic parameters including terminal $T_{1/2}$, clearance, volume of distribution at steady state, area under the concentration-time curve

and will be derived using noncompartmental analysis using Kinetica software version 4.3 (InnaPhase Corp., Philadelphia, PA).

**Study B. Optional subprotocol: To assess acetylation changes of histone proteins, induction of cell-cycle arrest or apoptosis, and activation of certain cellular genes in the pre- and post- treatment HCC tumor biopsies in association with PXD101**

The following tissues samples will be obtained:

a. Pre-treatment, either paraffin embedded tissues, or 4 mounted slide with unstained, unmelted, 5 micrometer thick sections containing adequate tumor, stored in room temperature.
b. Pre-treatment tumor biopsy tissues (stored at -80$^0$C until time for analysis).
c. Post treatment liver biopsy /fine needle aspirates from tumor tissues obtained after 2 cycles of study treatment (i.e. approximately 4-5 weeks after commencing study treatment). To be stored at -80$^0$C until time for analysis.

Please refer to Appendix F2, for sample collection form.

Shipping instructions of samples:

- A fax of the inventory sheet should be sent to the attention of Miss Jane Koh, Comprehensive Cancer Trials Unit, Room G05, G/F,Sir Y K Pao Cancer Centre, Prince of Wales Hospital, Shatin, NT, Hong Kong, the day before shipment of samples. The fax number is (852) 2947 8901; the tel number is (852) 2632 1142.
- Samples and inventory sheet must be shipped by overnight delivery in a styrofoam container and packaged in dry ice to ensure that they remain frozen. Shipment must be scheduled for weekdays only, and weekends will be avoided.

**SEND SAMPLES TO:** Attn: Miss Jane Koh,
Comprehensive Cancer Trials Unit,
Room G05, G/F,
Sir Y K Pao Cancer Centre,
Prince of Wales Hospital,
Shatin, NT,
Hong Kong

**Methods in brief:**

1. Hyperacetylation of histone proteins as described above.
2. Upregulation of certain cellular genes which are not methylated in tumors: p21$^{CIP1/WAF1}$, p27, p16 and ATM (Semi-quantitative RT-PCR; attempts on RT-PCR will be conducted)
3. Induction of cell-cycle arrest in HCC tumor cells will be assessed by flow cytometry.
4. Changes of apoptotic index in HCC tumor cells assessed will by TUNEL labeling on paraffin section.

**Study C. Optional subprotocol: Pharmacodynamic study: To assess epigenetic changes of peripheral blood in HCC patients during treatment with PXD101**

HCC has been detected to have aberrant promoter methylation in tumour and/or peripheral

blood and include *RASSF1A*, *p15, GSTP1* and *SFRP* (Wong 2000; Zhong 2002; Zhong 2003; Yeo 2004; Shih 2006). Further, in other tumor type, it has been shown that extent of promoter methylation can be altered with epigenetic therapy (Mirmohammadsadegh, 2006). These will be assessed in the peripheral blood of the studied patients. Blood samples will be collected before and following the start of infusion of cycle 1 of treatment, with the sampling schedule being:

**Day 1: 0, 1 h, 1.5 h, 2 h, 3 h, 5h, 24h**
**Day 5: 0, 1 h, 3h, 5h**
**Day 22: 0 min**

Venous blood samples should be collected in EDTA bottle. Centrifuge the EDTA tube at 2000 rpm for 5 min. Carefully remove the plasma with a disposable transfer pipette a transfer into a 5 ml tube. Store the plasma at -20C. For centres that require supply of bottles, please contact investigator in Charge: Dr Boon-Cher Goh.

Please refer to Appendix F3, for sample collection form.

Shipping instructions of samples:

- A fax of the inventory sheet should be sent to the attention of Ms Jane Koh Comprehensive Cancer Trials Unit, Room G05, G/F,Sir Y K Pao Cancer Centre, Prince of Wales Hospital, Shatin, NT, Hong Kong, the day before shipment of samples. The fax number is (852) 2947 8901; the tel number is (852) 2632 1142.
- Samples and inventory sheet must be shipped by overnight delivery in a styrofoam container and packaged in dry ice to ensure that they remain frozen. Shipment must be scheduled for weekdays only, and weekends will be avoided.

## 10. STUDY CALENDAR

***Schedules shown in the Study Calendar below are provided as an example and should be modified as appropriate.***

Baseline scans, x-rays and consent of patients must be done within 4 weeks prior to the start of therapy. Other baseline evaluations are to be conducted within 1 week prior to administration of protocol therapy. Prior to the first cycle, laboratory evaluations should be repeated within 72 hours prior to initiation of the study. For subsequent cycles, laboratory evaluations should be repeated within 72 hours prior to initiation of the next cycle of therapy.

| | Pre-Study | Wk 1 | Wk 2 | Wk 3 | Wk 4 | Wk 5 | Wk 6 | Wk 7 | Wk 8 | Wk 9 | Wk 10 | Wk 11 | Wk 12 | Off Study[d] |
|---|---|---|---|---|---|---|---|---|---|---|---|---|---|---|
| Treatment Cycle no. | | 1 | | | 2 | | | 3 | | | 4 | | | |
| PXD101[a] | | X | | | X | | | X | | | X | | | |
| Informed consent | X | | | | | | | | | | | | | |
| Demographics | X | | | | | | | | | | | | | |
| Medical history | X | | | | | | | | | | | | | |

| | | | | | | | | | | | | | | |
|---|---|---|---|---|---|---|---|---|---|---|---|---|---|---|
| Concurrent meds | X | X-------------------------------------------------------------------------------------------X | | | | | | | | | | | | |
| Physical exam | X | X | | | X | | | X | | | X | | | X |
| Vital signs | X | X | | | X | | | X | | | X | | | X |
| Height | X | | | | | | | | | | | | | |
| Weight | X | X | | | X | | | X | | | X | | | X |
| Performance status | X | X | | | X | | | X | | | X | | | X |
| CBC w/diff, plts, Prothrombin time | X | X | X | | X | X | | X | X | | X | X | | X |
| Serum chemistry[b] | X | X | X | | X | X | | X | X | | X | X | | X |
| ECG [g] | X | X | | | X | | | X | | | X | | | X |
| Adverse event evaluation | | X -----------------------------------------------------------------------------------------X | | | | | | | | | | | | X |
| Tumor measurements | X | Tumor measurements are repeated every 2 cycles of therapy. Documentation (radiologic) must be provided for patients removed from study for progressive disease. | | | | | | | | | | | | X[d] |
| Radiologic evaluation | X | Radiologic measurements should be performed every 2 cycles, approximately every 6 weeks. | | | | | | | | | | | | X[d] |
| B-HCG | X[c] | | | | | | | | | | | | | |
| AFP | X | | | | X | | | X | | | X | | | X |
| Pharmacokinetic study | | X[e] | | | X[e] | | | | | | | | | |
| Pharmacodynamic study | | X[h] | | | X[h] | | | | | | | | | |
| Optional Subprotocol | X[f] | | | | | | X[f] | | | | | | | |

a: PXD101: Dose as assigned; route/schedule.
b: Albumin, alkaline phosphatase, total bilirubin, BUN, calcium, creatinine, glucose, LDH, phosphorus, potassium, total protein, SGPT[ALT], sodium.
c: Serum or urine pregnancy test (women of childbearing potential).
d: Off-study evaluation.
e: Blood samples at the following time-point will be assessed: Day 1: 0, 15 min, 30 min, 45 min, 1 h, 1.5 h, 2 h, 3 h, 5h, 24h ; Day 5: 0, 30 min, 1 h, 1.5 h, 3h, 5h; Day 22: 0 min
f: Optional study: Pre- treatment tumor biopsies and post- treatment tumor biopsies/fine needle aspiration.
g: ECG monitoring should be done after completion of all PXD101 infusion , on day 1 and 5 of each cycle, to rule out QTc.
h: Optional study: Blood samples at the following time-point will be assessed: Day 1: 0, 1h, 1.5 h, 2 h, 3 h, 5h, 24h ; Day 5: 0, 1h, 3h, 5h; Day 22: 0 min

# 11. MEASUREMENT OF EFFECT

For the purposes of this study, patients should be reevaluated for response every 2 cycles of treatment (approximately 6 weeks). In addition to a baseline scan, confirmatory scans should also be obtained approximately 6 weeks (not less than 4 weeks) following initial documentation of objective response. Patients who have received a minimum of one cycle of treatment are evaluable for disease response, and response assessment should be done after the one cycle of treatment.

## 11.1 Definitions

Response and progression will be evaluated in this study using the new international criteria proposed by the Response Evaluation Criteria in Solid Tumors (RECIST) Committee [*JNCI* 92(3):205-216, 2000]. Changes in only the largest diameter (unidimensional measurement) of the tumor lesions are used in the RECIST criteria. Note: Lesions are either measurable or non-measurable using the criteria provided below. The term “evaluable” in reference to measurability will not be used because it does not provide additional meaning or accuracy.

### 11.1.1 Measurable disease

Measurable lesions are defined as those that can be accurately measured in at least one dimension (longest diameter to be recorded) as $\geq$20 mm with conventional techniques (CT, MRI, x-ray) or as $\geq$10 mm with spiral CT scan. All tumor measurements must be recorded in millimeters (or decimal fractions of centimeters).

### 11.1.2 Non-measurable disease

All other lesions (or sites of disease), including small lesions (longest diameter $<$20 mm with conventional techniques or $<$10 mm using spiral CT scan), are considered non-measurable disease. Bone lesions, leptomeningeal disease, ascites, pleural/pericardial effusions, lymphangitis cutis/pulmonis, inflammatory breast disease, abdominal masses (not followed by CT or MRI), and cystic lesions are all non-measurable.

### 11.1.3 Target lesions

All measurable lesions up to a maximum of five lesions per organ and 10 lesions in total, representative of all involved organs, should be identified as **target lesions** and recorded and measured at baseline. Target lesions should be selected on the basis of their size (lesions with the longest diameter) and their suitability for accurate repeated measurements (either by imaging techniques or clinically). A sum of the longest diameter (LD) for all target lesions will be calculated and reported as the baseline sum LD. The baseline

sum LD will be used as reference by which to characterize the objective tumor response.

#### 11.1.4 Non-target lesions

All other lesions (or sites of disease) should be identified as **non-target lesions** and should also be recorded at baseline. Non-target lesions include measurable lesions that exceed the maximum numbers per organ or total of all involved organs as well as non-measurable lesions. Measurements of these lesions are not required, but the presence or absence of each should be noted throughout follow-up.

### 11.2 Guidelines for Evaluation of Measurable Disease

All measurements should be taken and recorded in metric notation using a ruler or calipers. All baseline evaluations should be performed as closely as possible to the beginning of treatment and never more than 4 weeks before the beginning of the treatment.

The same method of assessment and the same technique should be used to characterize each identified and reported lesion at baseline and during follow-up. Imaging-based evaluation is preferred to evaluation by clinical examination when both methods have been used to assess the antitumor effect of a treatment.

**Clinical lesions.** Clinical lesions will only be considered measurable when they are superficial (e.g., skin nodules and palpable lymph nodes). In the case of skin lesions, documentation by color photography, including a ruler to estimate the size of the lesion, is recommended.

**Chest x-ray.** Lesions on chest x-ray are acceptable as measurable lesions when they are clearly defined and surrounded by aerated lung. However, CT is preferable.

**Conventional CT and MRI.** These techniques should be performed with cuts of 10 mm or less in slice thickness contiguously. Spiral CT should be performed using a 5 mm contiguous reconstruction algorithm. This applies to tumors of the chest, abdomen, and pelvis. Head and neck tumors and those of extremities usually require specific protocols.

**Ultrasound (US).** When the primary endpoint of the study is objective response evaluation, US should not be used to measure tumor lesions. It is, however, a possible alternative to clinical measurements of superficial palpable lymph nodes, subcutaneous lesions, and thyroid nodules. US might also be useful to confirm the complete disappearance of superficial lesions usually assessed by clinical examination.

**Endoscopy, Laparoscopy.** The utilization of these techniques for objective tumor evaluation has not yet been fully and widely validated. Their uses in this specific context require sophisticated equipment and a high level of expertise that may only be available in some centers. Therefore, the utilization of such techniques for objective tumor response should be restricted to validation purposes in reference centers. However, such techniques may be useful to confirm complete pathological response when biopsies are obtained.

**Tumor markers**. Tumor markers alone cannot be used to assess response. If markers are initially above the upper normal limit, they must normalize for a patient to be considered in complete clinical response.

**Cytology, Histology.** These techniques can be used to differentiate between partial responses (PR) and complete responses (CR) in rare cases (e.g., residual lesions in tumor types, such as germ cell tumors, where known residual benign tumors can remain).

The cytological confirmation of the neoplastic origin of any effusion that appears or worsens during treatment when the measurable tumor has met criteria for response or stable disease is mandatory to differentiate between response or stable disease (an effusion may be a side effect of the treatment) and progressive disease.

## 11.3 Response Criteria

### 11.3.1 Evaluation of target lesions

| | |
|---|---|
| Complete Response (CR): | Disappearance of all target lesions |
| Partial Response (PR): | At least a 30% decrease in the sum of the longest diameter (LD) of target lesions, taking as reference the baseline sum LD |
| Progressive Disease (PD): | At least a 20% increase in the sum of the LD of target lesions, taking as reference the smallest sum LD recorded since the treatment started or the appearance of one or more new lesions |
| Stable Disease (SD): | Neither sufficient shrinkage to qualify for PR nor sufficient increase to qualify for PD, taking as reference the smallest sum LD since the treatment started |

### 11.3.2 Evaluation of non-target lesions

| | |
|---|---|
| Complete Response (CR): | Disappearance of all non-target lesions and normalization of tumor marker level |
| Incomplete Response/ Stable Disease (SD): | Persistence of one or more non-target lesion(s) and/or maintenance of tumor marker level above the normal limits |
| Progressive Disease (PD): | Appearance of one or more new lesions and/or unequivocal progression of existing non-target lesions |

Although a clear progression of “non-target” lesions only is exceptional, in such circumstances the opinion of the treating physician should prevail, and the progression status should be confirmed at a later time by the review panel (or study chair).

Note: If tumor markers are initially above the upper normal limit, they must normalize for a patient to be considered in complete clinical response.

11.3.3 **Evaluation of best overall response**

The best overall response is the best response recorded from the start of the treatment until disease progression/recurrence (taking as reference for progressive disease the smallest measurements recorded since the treatment started). The patient's best response assignment will depend on the achievement of both measurement and confirmation criteria (see Sections 11.3.1 and 11.4.1).

| Target Lesions | Non-Target Lesions | New Lesions | Overall Response |
|---|---|---|---|
| CR | CR | No | CR |
| CR | Incomplete response/SD | No | PR |
| PR | Non-PD | No | PR |
| SD | Non-PD | No | SD |
| PD | Any | Yes or No | PD |
| Any | PD | Yes or No | PD |
| Any | Any | Yes | PD |

Note:

- X Patients with a global deterioration of health status requiring discontinuation of treatment without objective evidence of disease progression at that time should be classified as having "symptomatic deterioration." Every effort should be made to document the objective progression, even after discontinuation of treatment.

- X In some circumstances, it may be difficult to distinguish residual disease from normal tissue. When the evaluation of complete response depends on this determination, it is recommended that the residual lesion be investigated (fine needle aspirate/biopsy) before confirming the complete response status.

## 11.4 Confirmatory Measurement/Duration of Response

### 11.4.1 Confirmation

To be assigned a status of PR or CR, changes in tumor measurements must be confirmed by repeat assessments that should be performed at approximately 6 weeks (no less than 4 weeks) after the criteria for response are first met. In the case of SD, follow-up measurements must have met the SD criteria at least once after study entry at a minimum interval of at approximately 6 weeks (not less than 6-8 weeks) (see section 11.3.3).

### 11.4.2 Duration of overall response

The duration of overall response is measured from the time measurement criteria are met for CR or PR (whichever is first recorded) until the first date

that recurrent or progressive disease is objectively documented (taking as reference for progressive disease the smallest measurements recorded since the treatment started).

The duration of overall CR is measured from the time measurement criteria are first met for CR until the first date that recurrent disease is objectively documented.

#### 11.4.3 Duration of Stable Disease

Stable disease is measured from the start of the treatment until the criteria for progression are met, taking as reference the smallest measurements recorded since the treatment started.

### 11.5 Progression-Free Survival

Not applicable.

### 11.6 Response Review

Not applicable.

## 12. DATA REPORTING / REGULATORY CONSIDERATIONS

Adverse event lists, guidelines, and instructions for adverse event reporting can be found in Section 7.0 (Adverse Events: List and Reporting Requirements).

### 12.1 Data Reporting

#### 12.1.1 Method

This study will be monitored by the Clinical Data Update System (CDUS) version 3.0. Cumulative CDUS data will be submitted quarterly to CTEP by electronic means. Reports are due January 31, April 30, July 31, and October 31. *Instructions for submitting data using the CDUS can be found on the CTEP web site (http://ctep.cancer.gov/reporting/cdus.html).*

#### 12.1.2 Responsibility for Submissions

Study participants are responsible for submitting CDUS data and/or data forms to the Coordinating Center quarterly by 1 July 2005, 1 October 2005, 2 January 2006 and 1 April 2006 to allow time for Coordinating Center compilation, Principal Investigator review, and timely submission to CTEP (see Section 12.1.1.). For trials monitored by CTMS, the monthly data submission to CTEP

from Theradex should be copied to the Coordinating Center.

The Coordinating Center is responsible for compiling and submitting CDUS data to CTEP for all participants and for providing the data to the Principal Investigator for review.

## 12.2 CTEP Multicenter Guidelines

This protocol will adhere to the policies and requirements of the CTEP Multicenter Guidelines. The specific responsibilities of the Principal Investigator and the Coordinating Center (Study Coordinator) and the procedures for auditing are presented in Appendix B.

- The Principal Investigator/Coordinating Center is responsible for distributing all IND Action Letters or Safety Reports received from CTEP to all participating institutions for submission to their individual IRBs for action as required.
- Except in very unusual circumstances, each participating institution will order DCTD-supplied agents directly from CTEP. Agents may be ordered by a participating site only after the initial IRB approval for the site has been forwarded by the Coordinating Center to the CTEP PIO (*PIO@ctep.nci.nih.gov*) except for Group studies.

## 12.3 Clinical Trials Agreement (CTA)

The agent(s), supplied by CTEP, DCTD, NCI, used in this protocol is/are provided to the NCI under a Collaborative Agreement (CTA) between CuraGen Corporation [hereinafter referred to as ACollaborator(s)@] and the NCI Division of Cancer Treatment and Diagnosis. Therefore, the following obligations/guidelines, in addition to the provisions in the AIntellectual Property Option to Collaborator@ contained within the terms of award, apply to the use of Agent(s) in this study:

1. Agent(s) may not be used for any purpose outside the scope of this protocol, nor can Agent(s) be transferred or licensed to any party not participating in the clinical study. Collaborator(s) data for Agent(s) are confidential and proprietary to Collaborator(s) and shall be maintained as such by the investigators. The protocol documents for studies utilizing investigational agents contain confidential information and should not be shared or distributed without the permission of the NCI. If a copy of this protocol is requested by a patient participating on the study or patient's family member, the individual should sign a confidentiality agreement. A suitable model agreement can be downloaded from http://ctep.cancer.gov.
2. For a clinical protocol where there is an investigational Agent used in

combination with (an)other investigational Agent(s), each the subject of different collaborative agreements, the access to and use of data by each Collaborator shall be as follows (data pertaining to such combination use shall hereinafter be referred to as "Multi-Party Data".):

a. NCI must provide all Collaborators with prior written notice regarding the existence and nature of any agreements governing their collaboration with NIH, the design of the proposed combination protocol, and the existence of any obligations that would tend to restrict NCI's participation in the proposed combination protocol.

b. Each Collaborator shall agree to permit use of the Multi-Party Data from the clinical trial by any other Collaborator solely to the extent necessary to allow said other Collaborator to develop, obtain regulatory approval, or commercialize its own investigational agent.

c. Any Collaborator having the right to use the Multi-Party Data from these trials must agree in writing prior to the commencement of the trials that it will use the Multi-Party Data solely for development, regulatory approval, and commercialization of its own investigational agent.

3. Clinical Trial Data and Results and Raw Data developed under a collaborative agreement will be made available exclusively to Collaborator(s), the NCI, and the FDA, as appropriate. All data made available will comply with HIPAA regulations.

4. When a Collaborator wishes to initiate a data request, the request should first be sent to the NCI, who will then notify the appropriate investigators (Group Chair for Cooperative Group studies, or PI for other studies) of Collaborator's wish to contact them.

5. Any data provided to Collaborator(s) for phase 3 studies must be in accordance with the guidelines and policies of the responsible Data Monitoring Committee (DMC), if there is a DMC for this clinical trial.

6. Any manuscripts reporting the results of this clinical trial must be provided to CTEP for immediate delivery to Collaborator(s) for advisory review and comment prior to submission for publication. Collaborator(s) will have 30 days from the date of receipt for review. Collaborator shall have the right to request that publication be delayed for up to an additional 30 days in order to ensure that Collaborator's confidential and proprietary data, in addition to Collaborator(s)'s intellectual property rights, are protected. Copies of abstracts must be provided to CTEP for forwarding to Collaborator(s) for courtesy review as soon as possible and preferably at least three (3) days prior to submission, but in any case, prior to presentation at the meeting or publication in the proceedings. Press releases and

other media presentations must also be forwarded to CTEP prior to release. Copies of any manuscript, abstract, and/or press release/ media presentation should be sent to:

Regulatory Affairs Branch, CTEP, DCTD, NCI
6130 Executive Boulevard, Suite 7111
Rockville, MD 20852
FAX 301-402-1584
E-mail: anshers@ctep.nci.nih.gov

The Regulatory Affairs Branch will then distribute them to Collaborator(s). No publication, manuscript or other form of public disclosure shall contain any of Collaborator's confidential/proprietary information.

# 13. STATISTICAL CONSIDERATIONS

## 13.1 Study Design/Endpoints

Clinical Endpoints:
Phase I portion of the study:
Primary:
1. To determine dose limiting toxicities (DLT) and establish maximum tolerated dose (MTD) of PXD101 in patients with inoperable HCC.
Phase II portion of the study:
Primary:
1. To assess tumor response according to RECIST criteria

13.1.2 Phase I Portion
The objective of determining DLT and MTD as defined in Section 6 will be addressed by the Phase I portion of the study. For the Phase 1 study, a minimum of 6 patients and a maximum of 18 patients will be required for the determination of MTD.

13.1.2 Phase II Portion
The objective of assessing if the study drug is active with respect to tumor response will be addressed by the Phase II portion of the study.
Statistical Considerations
The primary endpoint of this phase II study is measured by objective response. We would be interested in the treatment if the response rate was greater than 5% and would consider anything less to be uninteresting. Therefore, we are setting the null hypothesis for the response rate at 5% versus an alternative hypothesis of a response rate of 20%.
The trial will be conducted in 2 stages. In the first stage, 12 patients [including 6 from the phase 1 who are treated at MTD] will be accrued. If there is no response, we will stop accrual and concluded that the study treatment is uninteresting. If we have 1 or more responses, then an additional 25 patients will be accrued. In the second stage with a total

of 37 patients, if we observed 3 or less responses we will consider the treatments to be uninteresting. The type I error for this two-stage design is 10% with 90% power. The average sample size given the null hypothesis is true is 23.5 with 54% chance of early termination.

### 13.2 Sample Size/Accrual Rate

Phase I portion: minimum no. of accrual = 6; maximum no. of accrual = 18.
Phase II portion: In the first stage, 12 patients will be accrued [including 6 from the phase 1 who are treated at MTD]. If there is no response, we will stop accrual and concluded that the study treatment is uninteresting. If we have 1 or more responses, then an additional 25 patients will be accrued.
The expected accrual rate will be 2 patients/month.

### 13.3 Stratification Factors

There will be no stratification.

### 13.4 Analysis of Secondary Endpoints

Please refer to section 13.1 and 13.2.

### 13.5 Reporting and Exclusions

13.5.1 **Evaluation of toxicity.** All patients will be evaluable for toxicity from the time of their first treatment with PXD101.

13.5.2 **Evaluation of response.** All patients included in the study must be assessed for response to treatment, even if there are major protocol treatment deviations or if they are ineligible. Each patient will be assigned one of the following categories: 1) complete response, 2) partial response, 3) stable disease, 4) progressive disease, 5) early death from malignant disease, 6) early death from toxicity, 7) early death because of other cause, or 9) unknown (not assessable, insufficient data). [Note: By arbitrary convention, category 9 usually designates the “unknown” status of any type of data in a clinical database.]

All of the patients who met the eligibility criteria (with the possible exception of those who received no study medication) should be included in the main analysis of the response rate. Patients in response categories 4-9 should be considered to have a treatment failure (disease progression). Thus, an incorrect treatment schedule or drug administration does not result in exclusion from the analysis of the response rate. Precise definitions for categories 4-9 will be protocol specific.

All conclusions should be based on all eligible patients. Subanalyses may then be performed on the basis of a subset of patients, excluding those for whom major protocol deviations have been identified (e.g., early death due to other reasons, early discontinuation of treatment, major protocol violations, etc.). However, these subanalyses may not serve as the basis for drawing conclusions concerning treatment efficacy, and the reasons for excluding patients from the analysis should be clearly reported. The 95% confidence intervals should also be provided.

**REFERENCES**

Calvet X, Bruix J, Gines P, Bru C, Sole M, Vilana R, et al. (1990) Prognostic factors of hepatocellular carcinoma in the west: a multivariate analysis in 206 patients. Hepatology 12:753-60.

Di Gennaro, E., F. Bruzzese, G. Romano*, et al.* (2003). Antitumor effect of histone deacetylase inhibitor SAHA alone and combined with cytotoxic drugs in human colorectal cancer cells. *Proc Am Assoc Cancer Res*. 44:A3636.

Finn, P., C. Watkins, R. Romero*, et al.* (2002). Discovery and structure-activity relationships of novel classes of histone deacetylase inhibitors. *Proc Am Assoc Cancer Res*. 43:A3672.

Finnin, M.S., J.R. Donigian, A. Cohen*, et al.* (1999). Structures of a histone deacetylase homologue bound to the TSA and SAHA inhibitors. *Nature*. 401:188-193.

Fleming TR. (1982) One sample multiple testing procedure for phase II clinical trials. Biometrics 38:143-151.

Glaser, K.B., M.J. Staver, J.F. Waring*, et al.* (2003). Gene expression profiling of multiple histone deacetylase (HDAC) inhibitors: defining a common gene set produced by HDAC inhibition in T24 and MDA carcinoma cell lines. *Mol Cancer Ther*. 2:151-163.

Investigator's Brochure. (2004). PXD101 Investigator's Brochure version 2.0 March 2004.

Johnson PJ. (2002) Hepatocellular carcinoma: is current therapy really altering outcome? Gut 51(4):459-462.

Johnstone, R.W. (2002). Histone-deacetylase inhibitors: Novel drugs for the treatment of cancer. *Nat Rev Drug Discov*. 1:287-299.

Jones, P.A., and S.B. Baylin. (2002). The fundamental role of epigenetic events in cancer. *Nat Rev Genet*. 3:415-428.

Kalvinsh, I., E. Loza, and I. Starchenkov. (2002). Discovery and structure-activity relationships of a novel class of histone deacetylase inhibitors. *Proc Am Assoc Cancer Res*. A3672.

Kelly, W.K., V. Richon, and T. Troso-Sandoval. (2001). Suberoylanilide hydroxamic acid (SAHA), a Histone Deacetylase inhibitor: Biologic activity without toxicity. *Proc Am Soc Clin Oncol*. 20:A344.

Lai EC, Fan ST, Lo CM, Chu KM, Liu CL, Wong J. (1995) Hepatic resection for hepatocellular carcinoma. An audit of 343 patients. Ann Surg 221:291-8.

Leung TW, Johnson PJ. (2001) Systemic therapy for hepatocellular carcinoma. Semin Oncol.

28(5):514-20.

Marks, P.A., V.M. Richon, and R.A. Rifkind. (2000). Histone deacetylase inhibitors: Inducers of differentiation or apoptosis of transformed cells. *J Natl Cancer Inst*. 92:1210-1216.

Mirmohammadsadegh A, Marini A, Nambiar S, Hassan M, Tannapfel A, Ruzicka T, Hengge UR. Epigenetic Silencing of the PTEN Gene in Melanoma. Cancer Res 2006; 66(13): 6546-6552.

Okuda K. (1980) Primary liver cancers in Japan. Cancer 45:2663-72.

Parkin DM, Stjernsward J, Muir CS. (1984) Estimates of the worldwide frequency of twelve major cancers. Bull World Health Organ 62:163-82

Pawarode A, Voravud N, Sriuranpong V, Kullavanijaya P, Patt YZ. (1998) Natural history of untreated primary hepatocellular carcinoma: a retrospective study of 157 patients. Am J Clin Oncol 21:386-91.

Plumb, J.A., P.W. Finn, R.J. Williams, *et al*. (2003). Pharmacodynamic response and inhibition of growth of human tumor xenografts by the novel histone deacetylase inhibitor PXD101. *Mol Cancer Ther*. 2:721-728.

Rahmani, M., C. Yu, Y. Dai, *et al*. (2003). Coadministration of the heat shock protein 90 antagonist 17-allylamino- 17-demethoxygeldanamycin with suberoylanilide hydroxamic acid or sodium butyrate synergistically induces apoptosis in human leukemia cells. *Cancer Res*. 63:8420-8427.

Richon, V.M., S. Emiliani, E. Verdin, *et al*. (1998). A class of hybrid polar inducers of transformed cell differentiation inhibits histone deacetylases. *Proc Natl Acad Sci U S A*. 95:3003-3007.

Shih YL, Shyu RY, Hsieh CB, Lai HC, Liu KY, Chu TY, Lin YW. Promoter methylation of the secreted frizzled-related protein 1 gene SFRP1 is frequent in hepatocellular carcinoma. Cancer 2006; 107(3): 579-590.

Wong IH, Lo YM, Yeo W, Lau WY, Johnson PJ. Frequent p15 promoter methylation in tumor and peripheral blood from hepatocellular carcinoma patients. Clin Cancer Res 2000 Sep; 6(9): 3516-3521.

Yoshida, M., M. Kijima, M. Akita, and T. Beppu. (1990). Potent and specific inhibition of mammalian histone deacetylase both *in vivo* and *in vitro* by trichostatin A. *J Biol Chem*. 265:17174-17179.

Yu, X., Z.S. Guo, M.G. Marcu, *et al*. (2002). Modulation of p53, ErbB1, ErbB2, and Raf-1

expression in lung cancer cells by depsipeptide FR901228. *J Natl Cancer Inst*. 94:504-513.

W Yeo, PKS Chan, WM Ho, B Zee, KC Lam, KIK Lei, ATC Chan, TSK Mok, JJ Lee, TWT. Leung, S Zhong, PJ Johnson. Lamivudine for the Prevention of Hepatitis B virus Reactivation in HBsAg Seropositive Cancer Patients Undergoing Cytotoxic Chemotherapy. *J Clin Oncol* 2004, 22(5): 927-934.

W Yeo, PKS Chan, S Zhong, WM Ho, JL Steinberg, JS Tam, P Hui, NWY Leung, B Zee, PJ Johnson. Frequency of hepatitis B virus reactivation in cancer patients undergoing cytotoxic chemotherapy: A prospective study of 626 patients with identification of risk factors. *Journal of Medical Virology* 2000, 62: 299-307.

Yeo W, Wong N, Wong WL, Lai PB, Zhong S, Johnson PJ. High frequency of promoter hypermethylation of RASSF1A in tumour and plasma of patients with hepatocellular carcinoma. Liver International 2004, 25: 266-272.

Yeo W, Lam KC, Zee B, Chan PSK, Mo FKF, Ho WM, Wong WL, Leung TWT, Chan ATC, Ma B, Mok TSK, Johnson PJ. Hepatitis B Reactivation in Patients with Hepatocellular Carcinoma Undergoing Systemic Chemotherapy. *Annuals of Oncology* 2004, 15: 1661-1666.

Zhong S, Tang MW, Yeo W, Liu C, Lo YM, Johnson PJ. Silencing of GSTP1 gene by CpG island DNA hypermethylation in HBV-associated hepatocellular carcinoma. Clinical Cancer Research 2002; 8: 1087-1092.

Zhong S, Yeo W, Tang MW, Wong N, Lai PB, Johnson PJ. Intensive Hypermethylation of the CpG Island of Ras Association Domain Family 1A (*RASSF1A*) in HBV-associated Hepatocellular Carcinomas. Clin Cancer Res. 2003 Aug 15;9(9):3376-82.

# APPENDIX A

## Performance Status Criteria

| ECOG Performance Status Scale | | Karnofsky Performance Scale | |
|---|---|---|---|
| Grade | Descriptions | Percent | Description |
| 0 | Normal activity. Fully active, able to carry on all pre-disease performance without restriction. | 100 | Normal, no complaints, no evidence of disease. |
| | | 90 | Able to carry on normal activity; minor signs or symptoms of disease. |
| 1 | Symptoms, but ambulatory. Restricted in physically strenuous activity, but ambulatory and able to carry out work of a light or sedentary nature (e.g., light housework, office work). | 80 | Normal activity with effort; some signs or symptoms of disease. |
| | | 70 | Cares for self, unable to carry on normal activity or to do active work. |
| 2 | In bed <50% of the time. Ambulatory and capable of all self-care, but unable to carry out any work activities. Up and about more than 50% of waking hours. | 60 | Requires occasional assistance, but is able to care for most of his/her needs. |
| | | 50 | Requires considerable assistance and frequent medical care. |
| 3 | In bed >50% of the time. Capable of only limited self-care, confined to bed or chair more than 50% of waking hours. | 40 | Disabled, requires special care and assistance. |
| | | 30 | Severely disabled, hospitalization indicated. Death not imminent. |
| 4 | 100% bedridden. Completely disabled. Cannot carry on any self-care. Totally confined to bed or chair. | 20 | Very sick, hospitalization indicated. Death not imminent. |
| | | 10 | Moribund, fatal processes progressing rapidly. |
| 5 | Dead. | 0 | Dead. |

## APPENDIX B

## CTEP MULTICENTER GUIDELINES

If an institution wishes to collaborate with other participating institutions in performing a CTEP sponsored research protocol, then the following guidelines must be followed.

Responsibility of the Protocol Chair

- The Protocol Chair will be the single liaison with the CTEP Protocol and Information Office (PIO). The Protocol Chair is responsible for the coordination, development, submission, and approval of the protocol as well as its subsequent amendments. The protocol must not be rewritten or modified by anyone other than the Protocol Chair. There will be only one version of the protocol, and each participating institution will use that document. The Protocol Chair is responsible for assuring that all participating institutions are using the correct version of the protocol.
- The Protocol Chair is responsible for the overall conduct of the study at all participating institutions and for monitoring its progress. All reporting requirements to CTEP are the responsibility of the Protocol Chair.
- The Protocol Chair is responsible for the timely review of adverse events to assure safety of the patients.
- The Protocol Chair will be responsible for the review of and timely submission of data for study analysis.

Responsibilities of the Coordinating Center

- Each participating institution will have an appropriate assurance on file with the Office for Human Research Protection (OHRP), NIH. The Coordinating Center is responsible for assuring that each participating institution has an OHRP assurance and must maintain copies of IRB approvals from each participating site.
- Prior to the activation of the protocol at each participating institution, an OHRP form 310 (documentation of IRB approval) must be submitted to the CTEP PIO.
- The Coordinating Center is responsible for central patient registration. The Coordinating Center is responsible for assuring that IRB approval has been obtained at each participating site prior to the first patient registration from that site.
- The Coordinating Center is responsible for the preparation of all submitted data for review by the Protocol Chair.
- The Coordinating Center will maintain documentation of adverse event reports. There are two options for adverse event reporting: (1) participating institutions may report directly to CTEP with a copy to the Coordinating Center, or (2) participating institutions report to the Coordinating Center who in turn report to CTEP. The Coordinating Center will submit adverse event reports to the Protocol Chair for timely review.

- Audits may be accomplished in one of two ways: (1) source documents and research records for selected patients are brought from participating sites to the Coordinating Center for audit, or (2) selected patient records may be audited on-site at participating sites. If the NCI chooses to have an audit at the Coordinating Center, then the Coordinating Center is responsible for having all source documents, research records, all IRB approval documents, NCI Drug Accountability Record forms, patient registration lists, response assessments scans, x-rays, etc. available for the audit.

<u>Inclusion of Multicenter Guidelines in the Protocol</u>

- The protocol must include the following minimum information:
  - The title page must include the name and address of each participating institution and the name, telephone number and e-mail address of the responsible investigator at each participating institution.
  - The Coordinating Center must be designated on the title page.
  - Central registration of patients is required. The procedures for registration must be stated in the protocol.
  - Data collection forms should be of a common format. Sample forms should be submitted with the protocol. The frequency and timing of data submission forms to the Coordinating Center should be stated.
  - Describe how adverse events will be reported from the participating institutions, either directly to CTEP or through the Coordinating Center.
  - Describe how Safety Reports and Action Letters from CTEP will be distributed to participating institutions.

<u>Agent Ordering</u>

- Except in very unusual circumstances, each participating institution will order DCTD-supplied agents directly from CTEP. Agents may be ordered by a participating site only after the initial IRB approval for the site has been forwarded by the Coordinating Center to the CTEP PIO.

**Appendix C. List of medications and substances known or with the potential to interact with selected CYP450 isoenzymes**

| Substrates | | Inhibitors | Inducers |
|---|---|---|---|
| **CYP3A isoenymes** | | | |
| acetaminophen | Lercanidipine | Amiodarone | Rifampacin |
| alfentanyl | lidocaine | Azithromycin | Phenobarbital |
| alprazolam | loratadine | Cimetidine | Phenytoin |
| amiodarone | lovastatin (NOT pravastatin) | Ciprofloxacin | Dexamethasone |
| amlodipine | methadone | Clarithromycin | phenylbutaton |
| astemizole | midazolam | Clotrimazole | carbamatepine |
| atorvastatin | nelfinavir | Delaviridine | Sulfinapyrazone |
| azithromycin | nifedipine | diethyl-dithiocarbamate | Barbiturates |
| buspirone | nisoldipine | diltiazem | Carbamazepine |
| cafergot | nitrendipine | erythromycin | Efavirenz |
| caffeine | odanestron (in part) | fluconazole | glucocorticoids |
| carbamazepine | pimozide | fluvoxamine | modafinil |
| cerivastatin | progesterone | gestodene | nevirapine |
| chlorpheniramine | propranolol | grapefruit juice | Phenobarbital |
| cisapride | quinidine | indinavir | Phenytoin |
| clarithromycin | quinine | interleukin-10 | Pioglitazone |
| cocaine | ritonavir | itraconazole | Rifampin |
| codeine | salmeterol | ketoconazole | St. John's wort |
| cyclosporine | saquinavir | mibefradil | troglitazone |
| dapsone | sildenafil | Miconazole | |
| dextromethorphan | simvastatin | mifepristone | |
| diazepam | sirolimus | nefazodone | |
| diltiazem | tacrolimus (FK506) | nelfinavir | |
| Disopyramide ergotamine | tamoxifen | norfloxacin | |
| erythromycin | taxol | norfluoxetine | |
| estradiol | terfenadine | ritonavir | |
| felodipine | testosterone | saquinavir | |
| fentanyl | trazodone | Troleandomycin | |
| finasteride | triazolam | verapamil | |
| haloperidol | verapamil | | |
| hydrocortisone | vincristine | | |
| indinavir | zaleplon | | |
| irinotecan | zolpidem | | |

| CYP2D6 isoenzyme | | | |
|---|---|---|---|
| amitriptyline<br>Codeine<br>desipramine<br>Flecainide<br>Fluoxetine<br>fluvoxamine<br>imipramine<br>metoprolol | nortriptyline<br>paroxetine<br>Propafenone<br>Propranolol<br>Statins — Simvastatin etc<br>timolol | Quinidine<br>Flecainide<br>propafenone | |

| CYP2C isoenzyme | | | |
|---|---|---|---|
| cycloguanil<br>mephenytoin,<br>methobarbital | Phenytoin<br>Proguanil<br>Tolbutamide Warfarin | Cimetidine<br>Amiodarone | |

## Appendix D. Child's-Pugh's grading for cirrhosis

| Measurements | 1 point | 2 points | 3 points |
|---|---|---|---|
| Bilirubin (umol/l) | 17-34 | 35-50 | > 50 |
| PT prolongation (secs) | 1-3 | 4-6 | > 6 |
| Albumin (g/l) | >35 | 28-34 | < 28 |
| Ascites | None | Slight | Moderate to severe |
| Encephalopathy | None | Grade 1 or 2 | Grade 3 or 4 |

Child's-Pugh's grading Class A = 5-6 points = 'minimal' hepatic impairment
Child's-Pugh's grading Class B = 7-9 points = 'moderate' hepatic impairment
Child's-Pugh's grading Class C = 10-15 points = 'severe' hepatic impairment

**Appendix E1. Eligibility Screening Checklist**

## HCC PXD101 ELIGIBILITY SCREENING CHECKLIST

Patient Initial: _______________ Date of Birth (DD/MM/YY): _______________

Written Consent On: ________________

### Inclusion Criteria

| | Yes | No |
|---|---|---|
| Histologically or cytologically confirmed inoperable hepatocellular carcinoma. | ☐ | ☐ |
| Measurable disease, defined as at least one lesion that can be accurately measured in at least one dimension (longest diameter to be recorded) as ≥20 mm with conventional techniques or as ≥10 mm with spiral CT scan. | ☐ | ☐ |
| Age ≥18 years. | ☐ | ☐ |
| Life expectancy of greater than 12 weeks. | ☐ | ☐ |
| ECOG performance status. 2 (Karnofsky. 60%). | ☐ | ☐ |
| Patients must have normal organ and marrow function as defined below:<br>• leukocytes ≥3,000/mcL<br>• absolute neutrophil count ≥1,500/mcL<br>• platelets ≥100,000/mcL<br>• total bilirubin ≤ 30 umol/l<br>• albumin ≥ 28 g/l<br>• ALT(SGPT) ≤ 5.0 X institutional upper limit of normal<br>• alkaline phosphatase ≤ 6 x ULN<br>• prothrombin time ≤ 4 sec<br>• creatinine ≤ 150 umol/l<br>• absence of clinical ascites | ☐ | ☐ |
| Women of child-bearing potential and men agree to use adequate contraception (hormonal or barrier method of birth control; abstinence) prior to study entry and for the duration of study participation. | ☐ | ☐ |
| Ability to understand and the willingness to sign a written informed consent document. | ☐ | ☐ |

Investigator's Signature: _________________ Date: ________________

# HCC PXD101 ELIGIBILITY SCREENING CHECKLIST

Patient Initial: _______________ Date of Birth (DD/MM/YY): _______________

## Exclusion Criteria

| | Yes | No |
|---|---|---|
| Prior chemotherapy within 4 weeks (6 weeks for nitrosoureas or mitomycin C) prior to entering the study or those who have not recovered from adverse events due to agents administered more than 4 weeks earlier. | ☐ | ☐ |
| Not receiving any other investigational agents. | ☐ | ☐ |
| Known brain metastases. | ☐ | ☐ |
| History of allergic reactions attributed to compounds of similar chemical or biologic composition to PXD101 | ☐ | ☐ |
| Not taken valproic acid, another histone deacytelase inhibitor, for at least 2 weeks prior to enrollment. | ☐ | ☐ |
| Uncontrolled intercurrent illness including, but not limited to, ongoing or active infection, symptomatic congestive heart failure, unstable angina pectoris, cardiac arrhythmia, or psychiatric illness/social situations that would limit compliance with study requirements. | ☐ | ☐ |
| Pregnant women; for women  nursing infants, breastfeeding should be discontinued if the mother is treated with PXD101. | ☐ | ☐ |
| HIV-positive patients on combination antiretroviral therapy | ☐ | ☐ |

Investigator's Signature: _________________ Date: ________________

## Appendix E2. Patient Registration Form

# PATIENT REGISTRATION FORM
# HCC PXD101

Please complete this form after the Informed Consent has been obtained and all screening evaluations have been performed and evaluated by the study investigator. Fax the completed form to the study manager at fax No: 852-2632 5816

**Date of Request:** ☐☐-☐☐☐-☐☐☐☐
(day) (month) (year)

**Section A: Patient and Investigator Information**

Principal Investigator: ______________________ Institution: __________________________
Contact Person:__________________________ Tel-No: _____________ Fax-No: _______________

Patient's Initials: ☐ ☐ ☐
first middle last

Patient's Date of Birth: ☐☐-☐☐☐-☐☐☐☐
(day) Month) (year)

**Section B: Inclusion/Exclusion Criteria**

Date informed consent signed. ☐☐-☐☐☐-☐☐☐☐
(day) (Month) (year)

Have all inclusion/exclusion criteria been confirmed and the patient is eligible for the study ☐ yes ☐ no

**Section C: Disease Related Issues/ Planned Treatment Date**

- Date of histological confirmation of primary diagnosis of HCC: ☐☐-☐☐☐-☐☐☐☐
(day) (Month) (year)

- Inoperable disease ☐ Yes ☐ No

Reason(s) for inoperable disease? (tick all that apply)
- ☐ Localized disease in a portion of the liver that does not allow the possibility of complete surgical removal of the tumour with a clear resection margin.
- ☐ Presence of extra-hepatic disease
- ☐ Main portal vein or hepatic vein involvement (invasion or tumour thrombus)
- ☐ Other, please specify ______________________________________

Patient is receiving medications/substances which may potentially which is a substrate/inducer/inhibitor of cytochrome P450 enzymes. ☐ Yes ☐ No

**Planned date of first treatment**: ☐☐-☐☐☐-☐☐☐☐
(day) (Month) (year)

**If you do not receive an Eligibility confirmation from the Sponsor within 24 hours of submission, please call your study monitor**

**Section E: Enrollment Authorization**

Patient Eligibility for study:

☐ **ELIGIBLE** Assigned Patient Number: ☐☐☐☐

☐ **NOT ELIGIBLE**

The patient is ineligible for the study. List patient in the screening log with the reason for ineligibility noted.

Reason for patient ineligibility/exclusion: ______________________________________________

Reviewed By: (Name printed) ____________________________

Signature: ____________________________ Date: ☐☐-☐☐☐-☐☐☐☐
(day) (Month) (year)

## Appendix F1. Sample data collection form: Pharmacokinetics

### SAMPLE DATA COLLECTION FORM: PHARMACOKINETICS HCC PXD101

Please complete this form in the appropriate section after each blood taking.

Patient's Study number: ☐☐☐☐☐ **Date:** ☐☐-☐☐☐-☐☐☐☐
(day) (month) (year)

**Section A: Patient and Investigator Information**

Principal Investigator: ____________________ Institution: ____________________
Contact Person:____________________ Tel-No: ______________ Fax-No: ______________

Patient's Initials: ☐ ☐ ☐ Patient's Date of Birth: ☐☐-☐☐☐-☐☐☐☐
first middle last (day) Month) (year)

Details of blood taking

| Date of blood taking | Timing of blood taking* | Blood taken (please delete as appropriate) | Serial no. | Sample no.** |
|---|---|---|---|---|
| (Day 1) | 0 min | Yes No | 01 | - |
| | 15 min | Yes No | 02 | - |
| | 30 min | Yes No | 03 | - |
| | 45 min | Yes No | 04 | - |
| | 1 h | Yes No | 05 | - |
| | 1.5 h | Yes No | 06 | - |
| | 2 h | Yes No | 07 | - |
| | 3 h | Yes No | 08 | - |
| | 5 h | Yes No | 09 | - |
| | 24 h | Yes No | 10 | - |
| (Day 5) | 96 h | Yes No | 11 | - |
| | 96 h 30 min | Yes No | 12 | - |
| | 97 h | Yes No | 13 | - |
| | 97 h 30 min | Yes No | 14 | - |
| | 99 h | Yes No | 15 | - |
| | 101 h | Yes No | 16 | - |
| | | | | |
| | | | | |

* This refers to the time from the first infusion of cycle 1 day 1
** Sample no. refers to the patient study no. followed by serial no. This should be labeled in each specimen bottle

## Appendix F2. Sample data collection form: Tumor tissue assessment

# SAMPLE DATA COLLECTION FORM: TUMOR TISSUE ASSESSMENT
# HCC PXD101

Please complete this form in the appropriate section after each blood taking.

Patient's Study number: ☐☐☐☐☐ **Date:** ☐☐-☐☐☐-☐☐☐☐
(day) (month) (year)

**Section A: Patient and Investigator Information**

Principal Investigator: ______________________ Institution: __________________________
Contact Person:__________________________ Tel-No: _____________ Fax-No: _______________

Patient's Initials: ☐ ☐ ☐ Patient's Date of Birth: ☐☐-☐☐☐-☐☐☐☐
first middle last (day) Month) (year)

Details of sample

| | Date of specimen | Fresh frozen/paraffin blocks/slides | Serial no. | Sample no.* |
|---|---|---|---|---|
| Pre-treatment | | | 01b | - |
| Post-treatment | | | 02b | - |
| | | | | |

* Sample no. refers to the patient study no. followed by serial no.; this should be labeled in each specimen bottle

## Appendix F3. Sample data collection form: Pharmacodynamics

### SAMPLE DATA COLLECTION FORM: PHARMACODYNAMIC
### HCC PXD101

Please complete this form in the appropriate section after each blood taking.

Patient's Study number: ☐☐☐☐☐ **Date:** ☐☐-☐☐☐-☐☐☐☐
(day) (month) (year)

**Section A: Patient and Investigator Information**

Principal Investigator: ________________________ Institution: ____________________________
Contact Person:____________________________ Tel-No: _______________ Fax-No: _________________

Patient's Initials: ☐ ☐ ☐ Patient's Date of Birth: ☐☐-☐☐☐-☐☐☐☐
first middle last (day) Month) (year)

Details of blood taking

| Date/Time of blood taking | Timing of blood taking* | Blood taken (please delete as appropriate) | Serial no. | Sample no.** |
|---|---|---|---|---|
| (Day 1______) | | | | |
| _ _: _ _ | 0 min | Yes No | PD01 | - |
| _ _: _ _ | 1 h | Yes No | PD02 | - |
| _ _: _ _ | 1.5 h | Yes No | PD03 | - |
| _ _: _ _ | 2 h | Yes No | PD04 | - |
| _ _: _ _ | 3 h | Yes No | PD05 | - |
| _ _: _ _ | 5 h | Yes No | PD06 | - |
| _ _: _ _ | 24 h | Yes No | PD07 | - |
| (Day 5______) | | | | |
| _ _: _ _ | 96 h | Yes No | PD08 | - |
| _ _: _ _ | 97 h | Yes No | PD09 | - |
| _ _: _ _ | 99 h | Yes No | PD10 | - |
| _ _: _ _ | 101 h | Yes No | PD11 | - |
| (Day 22_____) | | | | |
| _ _: _ _ | 0 min | Yes No | PD12 | - |

* This refers to the time from the first infusion of cycle 1 day 1

** Sample no. refers to the patient study no. followed by serial no. This should be labeled in each specimen bottle.

**APPENDIX G. Central Review of ECG tracings that are associated with QTc prolongation**

CuraGen has provided the following information regarding central reading of ECGs:

eResearch Technology, Inc. (eRT), the CuraGen reference lab, will send a manual to each site describing the logistics of collecting and sending EKGs. Briefly, the procedure is as follows:

In the event of a QTc reading >500 ms on a local machine, all available tracings will be submitted to eRT in Philadelphia, PA for a manual digital overread. Specific details will be provided to you in the form of an Investigator Guide directly from eRT. The process is generally as follows:

- Obtain all available original* ECG tracings from the study period for the patient.
- Conceal patient identification and affix study label completing requested information.
- Submit all available original* ECG tracings to eRT via DHL or FedEx. Account codes will be provided in the Investigator Guide.
- eRT will fax back analysis reports within 72 hours of their receipt. A sample report will be included in the Investigator Guide. Further dosing should be based upon the results of the central read as described in the letter.

*It is recommended that two original tracings are printed for each ECG. In cases where overread is needed, one original will be sent to eRT and the other will be retained as source document. If only one original tracing exists, the original tracing should be sent to eRT with a photocopy retained. The original will be returned following overread.
